# Coevolution of RNase P and the ribosome

**DOI:** 10.1073/pnas.2518495123

**Published:** 2026-03-02

**Authors:** Anton S. Petrov, Claudia Alvarez-Carreño, Loren Dean Williams, Mark A. Ditzler

**Affiliations:** ^a^National Aeronautics and Space Administration, Center for the Origins of Life, Georgia Institute of Technology, Atlanta, GA 30032; ^b^School of Chemistry and Biochemistry, Georgia Institute of Technology, Atlanta, GA 30032; ^c^Department of Structural and Molecular Biology, University College London, London WC1E 7JE, United Kingdom; ^d^Center for the Emergence of Life, National Aeronautics and Space Administration, Ames Research Center, Moffett Field, CA 94043

**Keywords:** RNA evolution, tranlsation, origin of life

## Abstract

Translation is carried out by the most conserved assemblies in biology. Among these assemblies, the ribosome and RNase P are central players. These ancient ribonucleoprotein complexes achieved structural and functional maturity by the last universal common ancestor (LUCA) of life. In prior work, we reconstructed the evolutionary history of the ribosome using its three-dimensional structure, based on accretion and molecular fingerprints that date back to life’s earliest stages. Here, we extend our structural phylogenetic framework—based on the accretion model—to RNase P, a ribonucleoprotein responsible for processing pre-tRNAs. By sampling RNase P RNA (RPR) sequences and structures across phylogeny and partitioning them into RNA fragments based on insertion fingerprints, we characterize the state of RPR at LUCA and reconstruct the chronology of its emergence. The chronology reveals that RNase P, like the ribosome, accreted modular RNA elements over evolution, while preserving the structure of preexisting elements, thus maintaining a structural record. We used interactions with tRNA to link and unify the evolutionary trajectories of RPR and rRNA. These results support the view that RNase P and the ribosome coevolved as part of a functionally integrated system. The ancestral catalytic sites of rRNA and RPR formed by the same process, fusion of two stem–elbow–stem elements. Analysis of these two coevolving RNAs also suggests that some of their accreted elements share common ancestry. Application of the accretion model requires correct secondary structures and was successful for RPR only when the traditional secondary structure was corrected by reorganizing a pseudoknot.

## Significance

RNase P is an ancient RNA–protein complex essential for tRNA maturation and thus protein translation. We present a model for the origins and evolution of RNase P. We incorporated phylogenetic data and 3D structures into the accretion formalism, where RNA evolves through stepwise addition of identifiable expansion segments. The results help visualize the emergence and development of RNase P at a molecular level. We deduce progression to a catalytic domain, which adds a specificity domain, then a conserved core, and finally lineage-specific expansions. We offer a molecular-level scenario for the coevolution of the ribosome, tRNA, and RNase P. Our findings reveal important steps in the emergence of life on Earth and patterns in RNA evolution, structure, and function.

Translation—the final step of the central dogma—is carried out by the most conserved assemblies in biology (1). As a keystone of cellular function, translation provides a rich source of information about the emergence of the molecular machinery of life and its early evolution (2–4). The ribosome, a key component of this system, achieved structural and functional maturity by the time of the last universal common ancestor (LUCA) (1). The structure of the ribosome provides a basis for a fine-grained chronology of rRNA evolution before LUCA. In previous works, we reconstructed the evolutionary history of the ribosome from its three-dimensional structure (4–7), tracing molecular records back to the earliest stages of evolution.

Ribonuclease P (RNase P), a ribonucleoprotein essential for tRNA maturation, is functionally integrated into the translation system and appears to be as ancient as the ribosome itself (8–12). Evolutionary studies have advanced ideas about how RNase P emerged (13) and have revealed correlations between acquisition and loss of RNA and protein over evolution (11). Other work has revealed how RNase P diversified near the root of the tree of life (14) and in specific lineages (12). Together, RNase P and the ribosome form a coordinated system central to gene expression. Here, we extend our structural phylogenetic approach to explore the early evolution of RNase P and its coevolution with the ribosome.

**Fig. 1.**
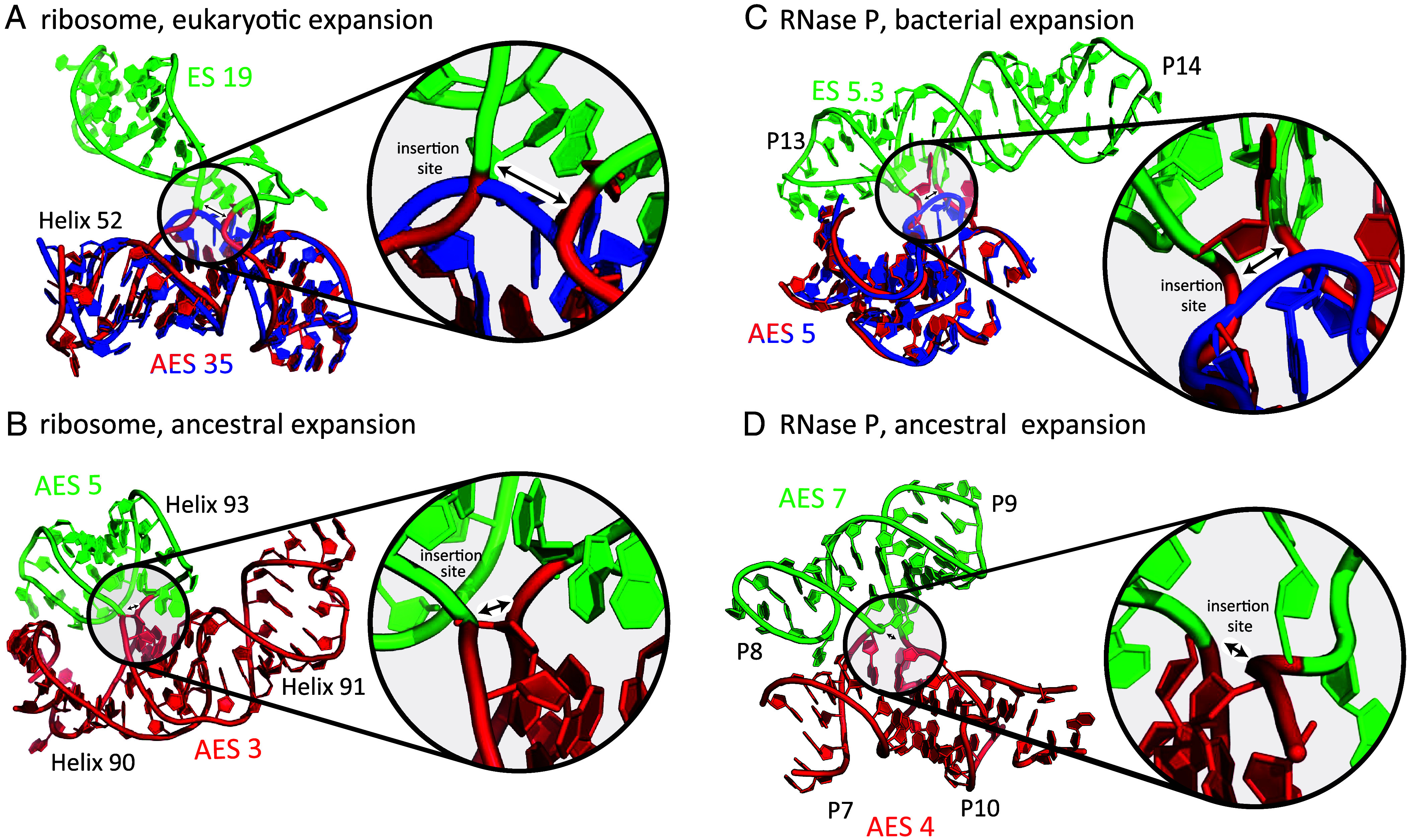
rRNA and RPR both contain insertion fingerprints that mark positions of expansions. (*A*) An insertion fingerprint indicates the known site of insertion of a eukaryotic expansion on the rRNA of the common core. The double-headed arrow indicates the site of expansion. rRNA of the common core is blue (*Escherichia coli*) (16) or red (*Saccharomyces cerevisiae*) (17). ES 19 is green. *S. cerevisiae* contains ES 19 and *E. coli* does not. (*B*) An rRNA insertion fingerprint indicates a site of expansion within the common core of the ribosome. The older AES is red, and the more recent AES is green. (*C*) An insertion fingerprint indicates the site of a bacterial expansion on the RPR common core. Common core RPR (AES5) is blue (*Bacillus subtilis*) (18) or red (*Thermotoga maritima*) (19). ES 5.3 is green (*T. maritima* only). (*D*) An insertion fingerprint within the common core of RPR (*T. maritima*). The older trunk AES 4 is red, and the more recent branch AES 7 is green. Labels of expansion segments match their colors; helical elements are labeled in black.

Our ancestral reconstructions of RNAs use the accretion model (6, 7). In this model, ancient RNAs evolved through sequential growth, with new segments added onto preexisting RNA scaffolds without altering underlying structure. Initial support for this model came from comparative analyses of three-dimensional structures of rRNAs, which revealed clear patterns of expansion over phylogeny. Sites of expansion are marked by characteristic structural features referred to as insertion fingerprints (Fig. 1*A*). Inserted lineage-specific RNA segments are referred to as expansion segments (ES). A key finding, allowing inference of deep evolution, was the observation of insertion fingerprints in the universally conserved ribosomal core. Their identification enabled characterization of ancestral expansion segments (AESs) (Fig. 1*B*), found within the common core, which predate LUCA. ESs and AESs are independent folding elements characterized by coaxial arrangement of base pairs with continuous stacking interactions and are separated from each other by insertion fingerprints (6, 15). ESs and AESs can be excised at the locations of the insertion fingerprints without perturbing the underlying structure.

Chronologies of AES incorporation are based on their topological connectivities, and the assumption that accretion proceeds as a continuous and recursive process. Chronologies are further refined using constraints derived from structural dependencies (20) between expansion segments (6), the accretion of AESs containing autonomous structural elements [e.g., RNA helices of A-minor motifs (21)] are inferred to precede AESs that are dependent on the autonomous structures (e.g., flipped adenines that interact within the minor grooves in A-minor motifs).

RNase P has much in common with the ribosome and is an attractive target for analysis by the accretion formalism. Like the ribosome, RNase P is an ancient multiple turnover ribozyme (22) that was mature and fully functional by LUCA (11, 23, 24). Like the ribosome, RNase P is a cog in the translation machinery. Like the core of rRNA, the core of RNase P RNA (RPR) is largely conserved across the tree of life in both secondary and three-dimensional structure (11, 25). Like in the ribosome, in RNase P, the protein components exhibit substantial variability across the tree of life (24, 26).

Here, we apply the accretion model to RPR and construct an evolutionary trajectory. AESs were defined and ranked into an accretion timeline based on the primary sequence, secondary structure, and three-dimensional structure of bacterial, archaeal, and eukaryotic RNase P (*SI Appendix*, Table S1, and FigShare Files S1 and S2 (27)). Special care was required in making these assignments in the context of a pseudoknot found in RPR (28), as its detection and annotation have historically been a source of ambiguity (29–31). We establish correlations between evolution of rRNA, tRNA, and RPR providing a detailed view into evolutionary processes, nearly 4 billion years ago, at the dawn of biology.

## Results

We applied the accretion formalism, which we used previously to describe the pre-LUCA evolution of rRNA (6, 7), to model the pre-LUCA evolution of RPR. We identified insertion fingerprints at sites of known, lineage-specific expansion (Fig. 1*C*) and within the common core of RPR (Fig. 1*D*). We partitioned RPR structures into AESs and ESs and identified universal and nearly universal AESs and clade-specific ESs. Finally, we assembled the resulting expansions, representing the evolution of RPR as a chronological accretionary process.

### The Traditional RPR Secondary Structure Is Incompatible With the Accretion Model.

**Fig. 2.**
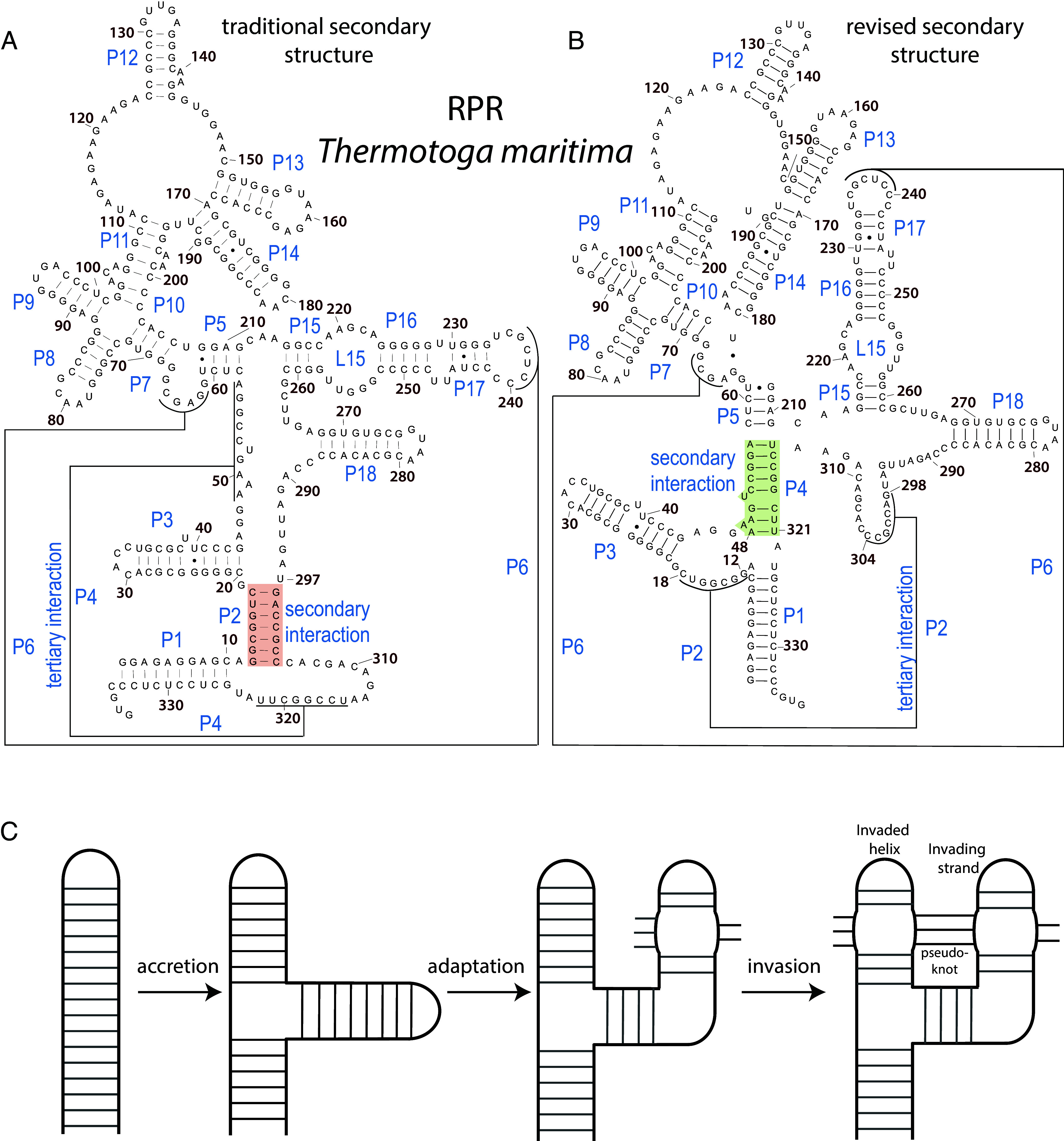
(*A*) The traditional secondary structure of the RNase P RNA (RPR) from *T. maritima*. In this structure, P2 is assigned as a secondary structural element (highlighted in red), while P4 is a tertiary structural element that forms a pseudoknot. (*B*) A revised secondary structure of the same RPR. In this model, P4 is reclassified as a secondary element (highlighted in green), and P2 is assigned as a tertiary element that forms a pseudoknot. Paired regions of RPR are labeled according to Ref. 8. The revised structure is consistent with experimental data on folding kinetics (29). (*C*) A schematic depiction of pseudoknot formation via accretion, adaptation, and strand invasion: A local duplex melts and one strand disrupts and invades another duplex, forming tertiary base pairs (a pseudoknot).

Using the traditional secondary structure (Fig. 2*A*) (8, 28), we partitioned RPR into expansion segments and attempted to establish a chronology of accretion. However, the traditional assignment of P2 as a secondary interaction and P4 as a tertiary interaction (pseudoknot) is incompatible with the accretion model (*SI Appendix,* Fig. S1). The resulting AES chronology violates continuity of accretion, forming entanglements of helices P1, P4, P5, and P15 (*SI Appendix,* Fig. S1*C*). The incompatibility of the traditional secondary structure of RPR with the accretion process is discussed further in the *SI Appendix*, *Text*.

A revision of the secondary structure of RPR, in which P4 is a secondary element and P2 is a tertiary element, removes the entanglement and is consistent with previous experimental results (29) and modeling studies (30). RPR folding kinetics demonstrated that P2 folds after P4. We therefore revised the secondary structure of RPR (Fig. 2*B*) and demonstrated that accretion of RPR is possible only within the framework of the revised secondary structure (*SI Appendix*, Fig. S1*B*).

### Reconstructing RPR at LUCA.

**Fig. 3.**
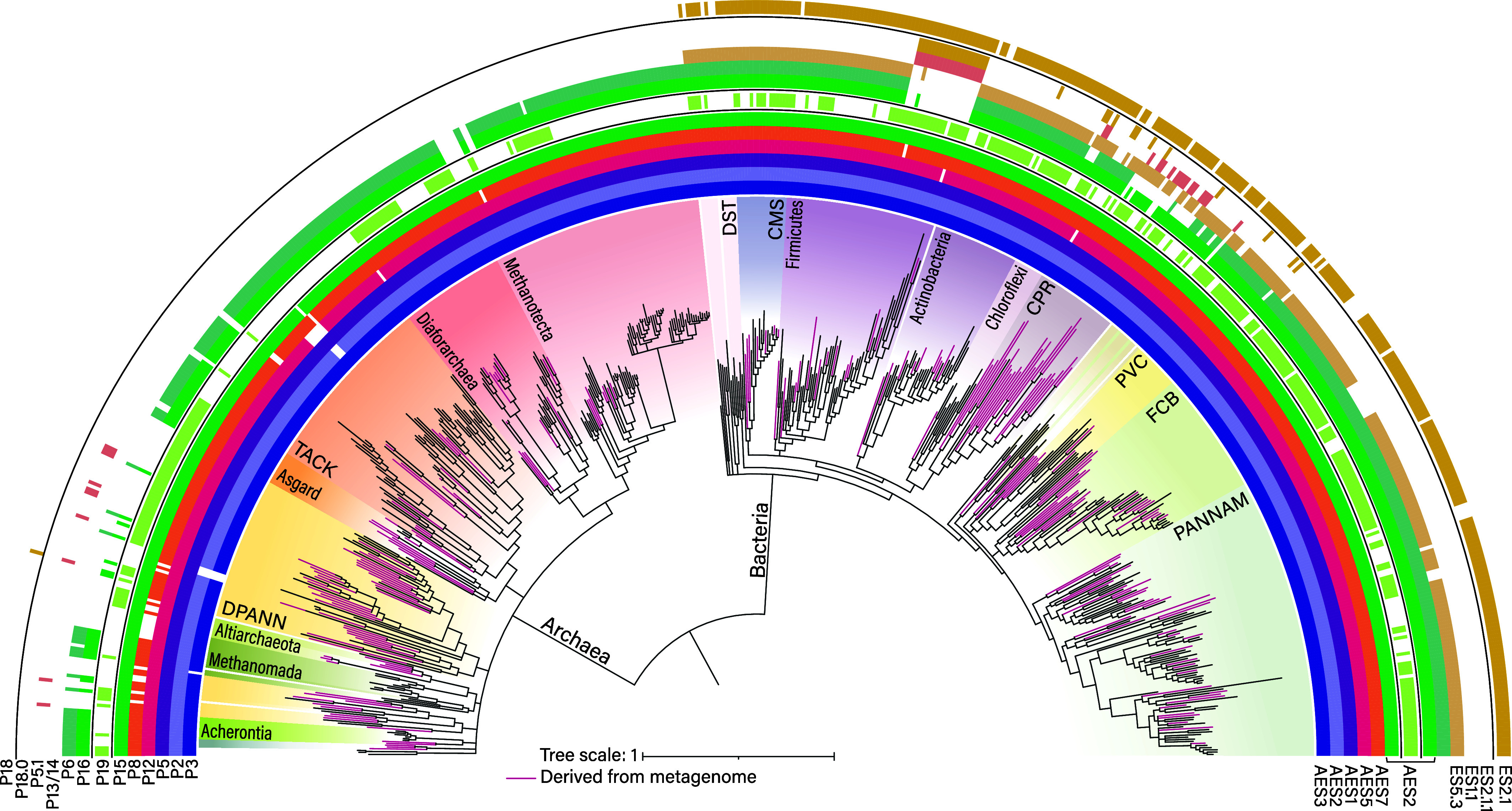
Phylogenetic distribution of RPR secondary structure elements. Secondary structure elements from 298 archaeal and 337 bacterial RPRs were mapped onto a maximum-likelihood tree of archaeal and bacterial lineages. Pink branches denote metagenome-derived sequences. Tree topology is adapted from Moody et al. (32). Helical elements are labeled on the *Left*, and the corresponding ESs and AES to which they belong are labeled on the *Right*. A mapping onto the tree with the complete topology across 349 archaeal and 350 bacterial species is shown in *SI Appendix,* Fig. S2.

We modeled the ancestral state of RPR at LUCA using secondary structure information inferred from complete RPR sequences of 298 archaeal and 337 bacterial species (Fig. 3 and *SI Appendix*, and FigShare Files S1–S4 (27)). This modeling also incorporated information from three-dimensional RPR structures from the three primary domains of life. We mapped secondary structural elements onto a previously constructed phylogenetic tree (32) derived from ribosomal protein sequences, analyzed the distribution of structural elements across lineages, and derived the most parsimonious estimate of RPR at LUCA. The helical elements within RPR at LUCA, which is composed of AESs (*SI Appendix,* Table S2), are either fully conserved in the archaeal and bacterial set (P1, P4, P5, P7, and P9) or are present in the majority of lineages across multiple phyla including deeply rooted species (P2, P3, P6, P8, P10, P11, P12, P16, P17, and P19). We also mapped lineage-specific helical elements.

No single RPR for which a 3D structure is known contains all the AESs in our model of RPR at LUCA. The closest representative structure is the RPR of *T. maritima* (3Q1Q). The *T. maritima* RPR lacks helix P19, which is represented in various clades of archaeal and bacterial species (Fig. 3), indicating its existence in the RPR of LUCA. However, P19 is present in RPR of *Geobacillus stearothermophilus* (2A64). Thus, the reconstruction of the RPR at LUCA is based on the 3D structures of RPR from *T. maritima* and *G. stearothermophilus.* P19 is of variable length over phylogeny and extends the stacking of pseudoknot P2.

### The Accretion Model of RPR.

The accretion model describes a phenomenological stepwise process of evolutionary growth of RNAs. The rules of the accretion model are agnostic to evolutionary framework, causality, driving force, function, and selective processes. The model of RPR evolution establishes temporal correlations between structure and function without building functional assumptions into the rules of the accretion process. While functional considerations are not used to build the accretion model, we do use the functions of structural modules in extant macromolecular complexes to infer their functions at the time of their incorporation into evolving RPR molecules.

**Fig. 4.**
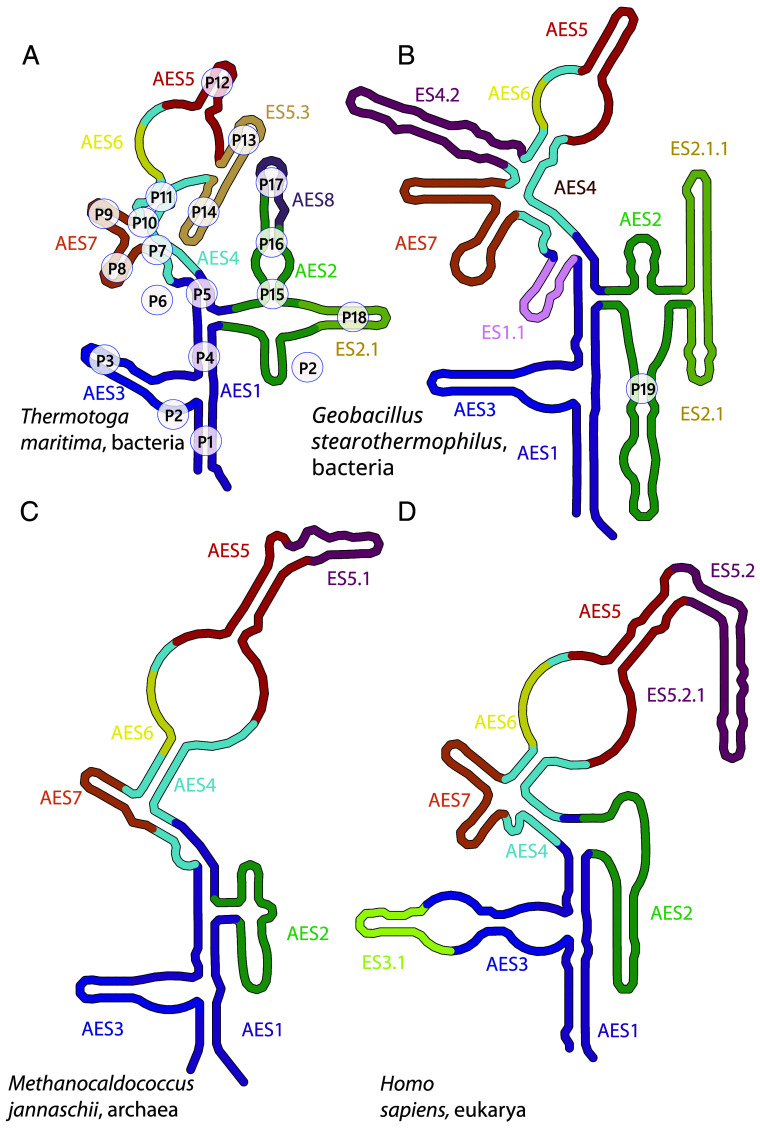
Revised secondary structures of RPRs, annotated with AESs and ESs. (*A*) *T. maritima* RPR, annotated with helices (paired regions). (*B*) *G. stearothermophilus* RPR (with P19 labeled, other helices as in panel *A*). (*C*) *Methanocaldococcus jannaschii* RPR. (*D*) *Homo sapiens* RPR. Each expansion segment is highlighted by a specific color. Additional secondary structures are provided in *SI Appendix*, Fig. S3, and FigShare File S6 (27).

**Fig. 5.**
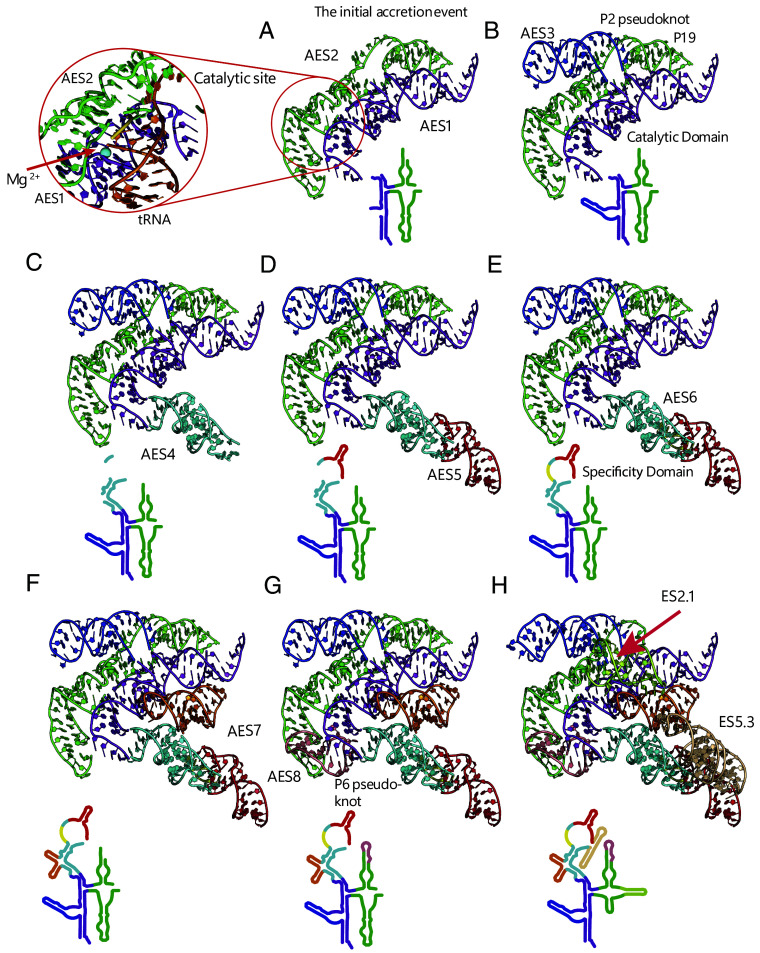
The accretion model of RPR emergence mapped onto the structures of RPRs of *T. maritima* (expansions AES1, AES4-AES8) and *G. stearothermophilus* (expansions AES2 and AES3). (*A*) Union of AES1 and AES2 forms the catalytic site of RPR, which is depicted in the inset in panel *A*. (*B*) Addition of AES3 integrates the catalytic domain by forming the pseudoknot P2. (*C*) the catalytic domain is extended by AES4, which interacts with tRNA’s TΨC-stem. (*D*) AES5 enhances interactions with tRNA. (*E*) Incorporation of AES6 results in the formation of a platform (together with AES5) that interacts with the tRNA elbow; catalytic and specificity domains are structurally integrated by (*F*) AES7 via A-minor interactions and (*G*) AES8 via an additional pseudoknot P6. (*H*) Alteration of the RPR core by lineage-specific *T. maritima* expansions and deletions. Expansion segments are highlighted in various colors using the coloring scheme in Fig. 4. The corresponding elements are also mapped onto the revised secondary structure of RPR and colored by matching the 3D colors of AESs. Discontinuities in the secondary structures indicate the locations of future insertions.

The accretion model, based on RPRs from the three domains of life, is depicted in Figs. 4 and 5. In this model, RPR started with the fusion of AES1 and AES2, a precursor of the full catalytic domain (18, 33, 34), which matured and expanded to ultimately acquire the specificity domain. Then, additional AESs contributed to overall stabilization. Specific steps in the accretion process are provided below as well as in *SI Appendix*, Table S3, and FigShare File S5 (27). After LUCA, RPR was elaborated with lineage-specific acquisitions of ESs and deletions throughout the RPR (*SI Appendix*, Table S4, and FigShare File S5 (27)). The RPR at LUCA is composed of AESs that form the catalytic core (AES1-AES3), the specificity domain (AES4-AES6), and AESs that reinforce the overall structural integrity of the assembly (AES7 and AES8).

### The Catalytic Domain.

The first accretion event involved the joining of AES1 and AES2 (Fig. 5*A*), two preexisting stem-elbow-stem motifs (35). Their association established the seminal RNA ancestor of RNase P. This RNA contained structural elements essential for catalysis and binding of the acceptor stem with the 3′ CCA tail and 5′ leader of pre-tRNA.

AES1 is composed of coaxially stacked helices P1, P4, and P5 (Figs. 2*B*, 4*A*, and 5*A*). AES1 is the most conserved element of RNase P. Unlike all other AESs, it is not lost or substantially reduced in any known RPR sequence (Figs. 3 and 4; *SI Appendix*, Fig. S2, and FigShare Files S3 and S6 (27)), and is highly conserved in sequence (*SI Appendix*, Fig. S4). In the extant RPR, AES1 participates in catalysis by coordinating catalytic metal ions at the active site as determined by both functional (36, 37) and structural (19, 38) studies, and it binds to the acceptor stem of the pre-tRNA (positions 1 to 3 of mature tRNA numbering), directly adjacent to the cleavage site. AES2 is composed of helices P15 and P16, one strand of P2, and helix P19. These three regions of AES2 are contained within a single coaxial structure with nearly continuous base stacking (Fig. 5*A*). Coaxial stacking within AES2 suggests that in its original form it was a continuous double helical element, containing P15 and P19. AES2 interacts with the 5′ leader of pre-tRNA (Fig. 5*A*, inset), and includes the structural element J5/15, which stacks on the terminal base pair of the acceptor stem. AES2 also encompasses Loop 15, which contributes to substrate recognition by binding to the pre-tRNA 3′ CCA tail (9, 19, 39). A construct containing Loop 15 with helices P15 and P16 has also been shown to support catalysis in vitro when isolated from the rest of the RPR (9), demonstrating its potential to contribute directly to catalysis along with P4 (19, 38), although Loop 15 is not essential for catalytic activity in the extant RNase P (40, 41). Thus, with the catalytic and recognition elements of extant RPR localized within AES1 and AES2, we infer that the accretion of these stem-elbow-stem fragments generated a (functional) synergy; either by establishing the initial catalytic function, or by enhancing preexisting catalytic function of the individual AESs.

In the accretion model, insertion of AES3 leads to the formation of pseudoknot P2, which integrates the core of the catalytic domain. AES3 accretes into AES1, then adapts and invades AES2 to form P2, which contains base pairs between nts 298 to 304 (AES2) and 12 to 18 (AES3), using the numbering scheme of *T. maritima* (Fig. 2*B*) (31). The pseudoknot is critical for shaping evolution of the catalytic domain of RNase P and helps explain its overall architecture by fostering coaxial stacking interactions between P3 and P19 and disrupting the stacking continuity between P15 and P19 (*SI Appendix*, *Text*).

A pseudoknot contains a tertiary interaction and cannot form through accretion alone; its formation requires an initial accretion event followed by remodeling of base-pairing interactions (Fig. 2*C*). We previously described a similar multiple-step mechanism of pseudoknot formation within rRNA (6, 7). After accretion, the incoming ES or AES (the invader) adapts its conformation, interacts with and ultimately disrupts another helix (the invaded) and pairs with one strand of it. The resulting pseudoknot is characterized by a tertiary helix between the invader and the invaded (schematically depicted in Fig. 2*C*). For example, in the 18S rRNA, part of es6 accretes then invades es3 and pairs to form a pseudoknot. This pseudoknot stabilizes the body region (42, 43) of the SSU and integrates the 5′ and C domains. Differentiation of secondary and tertiary interactions is key to correct definition of expansion segments but is sometimes nontrivial.

Our AES definition of the catalytic domain here is in essential agreement with the experimental data. This domain contains all elements that directly participate in catalysis of pre-tRNA cleavage. The catalytic domain interacts only with the acceptor stem of tRNA and lacks many recognition elements of mature RPR (*SI Appendix,* Fig. S5). Structural elements in AES1 and AES2 make independent contributions to both substrate recognition and catalysis, with AES3 integrating these elements.

### The Specificity Domain.

RPR underwent an additional series of expansions that enhanced its recognition of pre-tRNA (33, 44, 45). AES4 accretes onto AES1, embracing the TΨC-stem of pre-tRNA. AES4 is formed by the coaxially stacked helices P7, P10, and P11, and makes a sharp turn from the stacking interactions in AES1. AES4 is elaborated by AES5, which extends beyond the elbow of pre-tRNA and interacts with the T-loop (Fig. 5*D*). The interaction between AES4/AES5 and the T-loop of pre-tRNA is enhanced by accretion of AES6 (Fig. 5*E*), which is structurally dependent on preexisting AES5. AES6 is a small protrusion (patch) at the junction between AES4 and AES5, that forms tertiary base pairs with AES5. AES6 and AES5 form a highly conserved motif that interacts with the tRNA elbow via the stacking interactions with the G19:C56 platform of tRNA. The AES5/AES6 module is nearly identical to a module within the L1 stalk of LSU rRNA (45) (*SI Appendix*, Fig. S6 and *Text*).

### Integration of the Domains.

AES7 and AES8 integrate the cores of the specificity and catalytic domains, providing global stability to RPR. AES7 accreted onto AES4 (Fig. 5*F*), where it bridges the specificity and catalytic domains by interacting with AES1 by two A-minor interactions: i) between the terminal loop of P9 and the minor groove of P1 (11, 46), and ii) between the terminal loop of P8 and the minor groove of P4 (*SI Appendix,* Fig. S4 and Table S6). AES7 of some species (as seen in the *M. jannaschii* structure and inferred for *G. stearothermophilus*) forms contacts with pre-tRNA elbow via helix P9.

AES8 extends AES2, making a sharp turn and forming tertiary base pairing contacts (invading the boundary of AES1 and AES4, forming pseudoknot P6) and providing additional structural integrity of AES1 and AES2 near the catalytic site (Fig. 5*G*). We note that while chronologically AES1-6 appear in a linear progression as specified above by their indices, the chronology of emergence of AES7 and AES8 is not well constrained except that neither of them could appear before AES4. With the accretion of AES7 and AES8, the universal core of RPR was established.

### Evolution of RNase P Beyond LUCA.

Like that of rRNA, the common core of RPR (*SI Appendix,* Fig. S7*A*) grew by acquisition of AESs and was elaborated by lineage-specific acquisition of ESs. Unlike the rRNAs, RPR underwent significant deletions in many lineages. In bacterial RNase P, the RPR evolved to be larger in size, on average (*SI Appendix,* Fig. S8), than the archaeal and eukaryotic versions, while the archaeal and eukaryotic versions incorporated several additional proteins resulting in larger overall RNP complexes (11, 12). In some bacterial lineages, deletions within the RPR core were tolerated due to the presence of ESs that compensate for losses (e.g. within the *Bacilli-Mollicute* lineage described below), whereas deletions to RPR in the archaeal/eukaryotic lineage appear to have been tolerated because of the presence of multiple RNase P proteins that accumulated in those lineages.

### Variable Expansions and Deletions in Bacteria.

Lineage-specific expansion segments, ES2.1 and ES5.3, are broadly distributed across most bacteria (Figs. 3 and 5*H*) and were likely present in the RPR of the last bacterial common ancestor (LBCA). Accretion of ES2.1 introduced P18, which provides interdomain stabilization between the catalytic and specificity domains by forming an A-minor interaction with P8 in AES7 (*SI Appendix,* Table S6). Similarly, ES5.3 introduced P14, which provides intradomain stabilization within the specificity domain via an A-minor interaction also with P8. Additional expansions, which occurred post-LBCA, include ES1.1 (P5.1), ES2.1.1 (P18.0) (*SI Appendix,* Fig. S9), and ES4.2 (P10.1) (Figs. 3 and 4*B* and *SI Appendix,* Table S4). All of these expansions were accreted distal to the RPR–tRNA interface (*SI Appendix,* Fig. S7*B*), where they stabilize the RPR structure, but do not interact directly with the substrate.

Several bacterial lineages have lost RPR elements present at LUCA. These deletions often coincide with and were likely facilitated by the addition of ESs. For example, in the *Bacilli-Mollicute* lineage, all of AES8 and part of AES2 are lost. This deletion resulted in the loss of the P6 pseudoknot, which formed a structural bridge from AES2 in the catalytic domain to the boundary of AES1 and AES4 at the interdomain junction. This deletion coincided with the accretion of P18.0 as part of ES 2.2.1 and P5.1 as part of ES1.1 (Fig. 4*B*). A tertiary interaction between P18.0 and P5.1 provides an alternative structural bridge between the catalytic domain and the interdomain junction, which likely facilitated the loss of P6 in this lineage. Several additional deletions are observed among bacterial RPRs, including multiple, independent losses of P6 and P19 (*SI Appendix*, *Text*).

### Variable Deletions and Expansions in Archaea and Eukarya.

RPR underwent extensive proteinization in the archaeal/eukaryotic lineage, compared to in the bacterial lineage. The last archaeal common ancestor (LACA) acquired five proteins (Rpp21, Rpp29, Rpp30, Pop5, and Rpp38; *SI Appendix,* Fig. S7*C*) (41), with additional proteins appearing later within the eukaryotic branch (47). These proteins provide structural stabilization to the RPR and are located on the proximal side of RPR (where tRNA binds), where four of them (Rpp21, Rpp29, Rpp30, Pop5) interact directly with the substrate (*SI Appendix,* Fig. S7*D*). The five archaeal proteins were likely exapted into RNase P structure from homologs that emerged during the evolution of translation and transcription machineries at the origins of LACA (*SI Appendix*, *Text* and Fig. S10). The acquisition of proteins at LACA may have provided overall stability and robust performance to the archaeal RNase P, enabling deletions in RPR. These deletions occurred independently along evolutionary trajectories across multiple archaeal lineages. As with bacteria, AES8 and AES2 are frequent sites of deletion resulting in the loss of P6, but these deletions are generally much more substantial compared to bacteria, often including all of P16 and P17 (Figs. 3 and 4*C*), and in some cases all of P15 (Figs. 3 and 4*D*). Unlike in bacteria, these deletions in general do not coincide with the additions of ESs, and are likely tolerated due to the presence of RNase P proteins (41), which provide alternative mechanisms for structural stabilization (11). As with bacterial lineages, P19 is sporadically lost among the Archaea and eukaryotes. In some organisms (species of *Halobacterium* class within *Methanotecta* superclass of Archaea) loss of P19 coincides with enhanced growth in P12.1/P12.2 (*SI Appendix*, Fig. S11). Additional deletions include P3 from AES3, P2 from AES2 observed within *Nanoarchaeales* (*Nanopusillaceae*) (*SI Appendix,* Fig. S12), and P8 from AES7 (Figs. 3 and 4*C*). In extreme cases observed within *Thermoproteaceae*, most of the specificity domain has been lost, including the entirety of AES5 and AES6 (48).

Although reduction is common in the evolution of archaeal/eukaryotic RPRs, expansions are also observed. Common expansions include ES5.1 and ES5.2 (Figs. 4 *C* and 4 *D*), which are frequently inserted into the tip of P12; and ES 3.1, which introduces an internal loop and an additional helix to the end of P3 (Fig. 4*D*). In both cases, these insertions provide additional binding sites for RNase P proteins (40, 41, 49).

## Discussion

We describe an evolutionary model of the origins and evolution of the RNA of RNase P (RPR). In this model, RPR originates as a primitive RNA that gains mass by accretion, and matures to a near-final RPR catalytic domain. The mature catalytic domain incrementally acquires the specificity domain. The two domains are further elaborated to integrate the overall structure forming the RPR common core. The accretion hierarchy is consistent with the experimental observations of independent folding of the two domains (38, 50). Together with related findings, this model allows us to begin reconstructing the coevolution of RNase P, tRNA, and the ribosome. Our approach is based on information drawn from sequences, secondary and three-dimensional structures obtained from organisms across the tree of life (4–7).

Key findings here are: i) RPR expanded by accretion during pre-LUCA evolution; ii) the common core was built by accretion of Ancestral Expansion Segments (AESs) which were recursively inserted without remodeling basal structures; iii) sites of AES insertion are marked in three-dimensions by characteristic fingerprints; iv) insertion chronology is reflected in structural dependencies; and v) RPR, unlike rRNA, experienced post-LUCA deletions in some lineages. We infer significant similarities and important differences in the histories of rRNA and RPR.

### RNase P, tRNA, and Ribosome Coevolution.

**Fig. 6.**
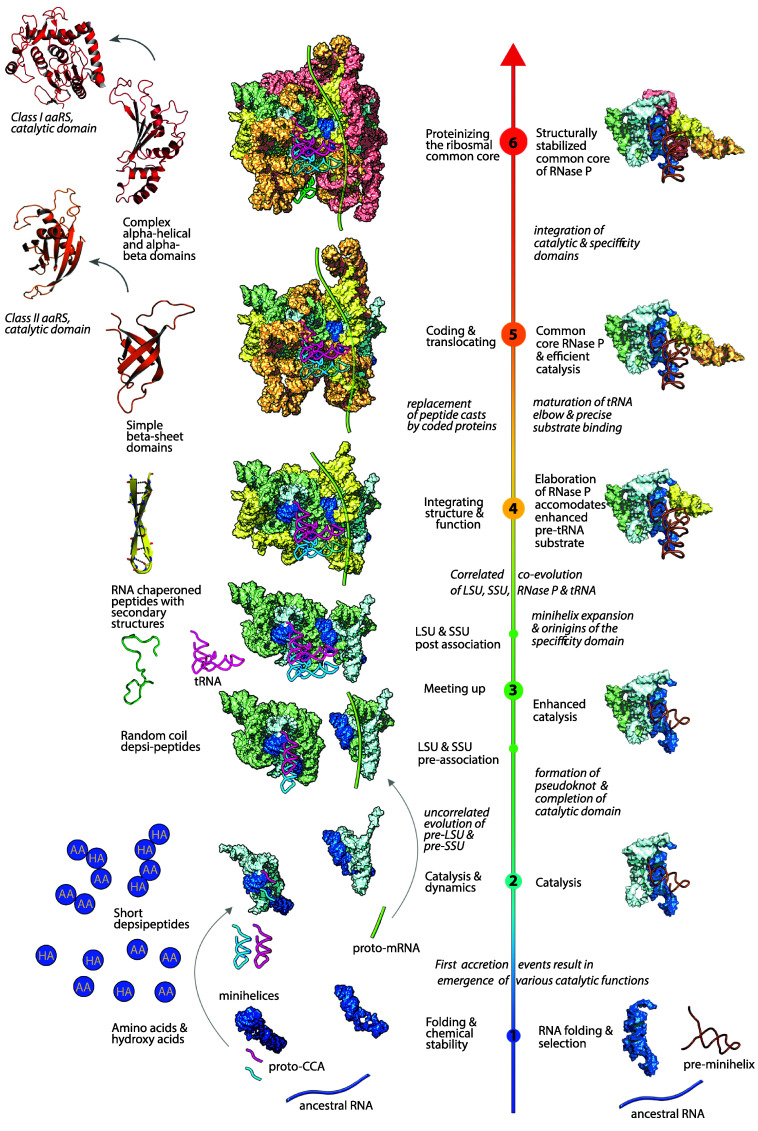
Unified chronology of evolution of RNase P, LSU and SSU rRNAs, and rProteins. The AESs of RPR, LSU, and SSU rRNAs were grouped into six corresponding phases as inferred from the accretion model and interactions of these complexes with tRNA (*SI Appendix,* Fig. S5). Correlations are indicated across RNA (7) and protein (5) components. A portion of this Figure was previously published in ref. 7 and is excluded from the Creative Commons license of this article.

We used tRNA as a molecular clock to link and unify the evolutionary trajectories of RPR, tRNA, and rRNA. tRNAs form direct molecular interactions with both RPR and rRNA. The evolution of those interactions provides chronological reference points for expansion of both RPR and rRNA. The underlying assumption is that the sequential acquisition of interactions with tRNA occurred concurrently in RPR and rRNA (Fig. 6 and *SI* *Appendix,* Fig. S5).

As described previously (7), the ribosomal common core evolves in six phases, each of which involves insertions of many AESs. The unified rRNA/tRNA/RPR model here suggests commonalities and correlated evolution at the level of phases. The accretion model begins with a selection of folding competent precursors of these molecules (minihelices or stem-elbow-stem fragments) from the pool of short RNA fragments based on their folding and metal binding affinities that enhanced resistance to RNA degradation or due to the emergence of catalytic activity (phase 1). The coevolutionary model of RPR, tRNA, and rRNA posits early recognition of a tRNA minihelix by RPR and rRNA catalytic ancestors (phases 2 and 3) (7, 51, 52). Early catalysis is followed by accretion of the anticodon stem onto the tRNA minihelix to form a boomerang-like proto-tRNA (53) (phase 4). Additional elaboration of tRNA involves maturation of the D and T loops to form the contemporary structure with a characteristic elbow, which structurally integrates the acceptor and anticodon stems (phase 5). The relationship between RPR AESs and phases as well as their color representations are provided in *SI Appendix*, Table S3 and Fig. S13.

The ancestral catalytic sites of rRNA and RPR form by a common process of fusion of two stem-elbow-stem elements (Phase 2) (52, 54). Catalytic sites of both RPR and rRNA interact with the 3′ tail and acceptor stem (minihelix) of proto-tRNA (Fig. 6, phases 2 & 3). Contacts of later rRNA and RPR states (phase 4) are with the TΨC-stem of tRNA. And finally, contacts with the tRNA elbow are only present in the mature states of rRNA and RPR (phase 5).

The accretion models for rRNAs and RPR are generally self-consistent and are supported by previous observations that both RNase P (51) and the ribosome (55) can use simple minihelices composed of an acceptor stem and 3′ tail as substrates. In the earliest stages (Phases 1 to 3), expansions of RPR and rRNA may not be tightly coupled. These phases represent accretion events within individual LSU, SSU, and RNase P systems, before acquisition of the subunit interface of the ribosome. During the later phases (Phases 4 to 6), the coevolution of all three RNA components of the translation machinery may have been tightly correlated.

A striking example of this correlation is provided by the emergence of an identical tRNA-elbow recognition motif in RPR and rRNA (44, 45). It is located within the L1 stalk of LSU rRNA and within the specificity domain of RPR (AES5 & 6). In both, this motif is used to recognize the tRNA elbow. It positions and stabilizes the elbow of the E-site tRNA in rRNA and the elbow of pre-tRNA in RPR (*SI Appendix*, Fig. S6). It stacks on the tRNA elbow through the same G19:C56 platform in both RPR and rRNA. The reverse evolutionary process (degeneration and disintegration of these components) is observed in mitochondria. tRNA of metazoan mitochondria lacks the elbow (56). The loss of the tRNA elbow coincides with the complete loss of the tRNA-elbow recognition motifs via i) reductions of L1 stalk of LSU, and ii) complete replacement of RPR with an evolutionarily unrelated, fully proteinaceous form (57). This correlation provides additional support for coupling of evolution between rRNA, tRNA, and RNase P.

The presence of a common structural solution for tRNA elbow recognition in RPR and rRNA is not easily explained by chance or convergence. We suggest that the elbow recognition motif arose once and was transferred—either from RPR to rRNA, from rRNA to RPR, or from a common ancestral source. This single-origin hypothesis of the elbow recognition motif is supported by several observations: The motif is large and complex, it appears nearly simultaneously in both rRNA and RPR, it performs essentially the same function in RPR and rRNA; and RNA elements are mobile (58) and commonly inserted into larger RNAs. In light of these considerations, a previously proposed model that the elbow recognition motif arose independently in rRNA and RPR through convergent evolution (44, 45) appears less plausible.

### Differential Reduction RPR and rRNA Evolution.

The results here indicate that RPR and rRNA evolved by similar precepts. Like rRNA (6, 7), RPR expanded over evolution by accretion, developed a common core that is generally conserved over the tree of life, and retained evidence of ancestral structure and function. However, the model also demonstrates remarkable differences between RPR and rRNA. The common RPR core is more malleable over evolution than rRNA. rRNA contains essentially all ribosomal AESs in nearly all lineages, except small deletions in some obligate pathogens. In contrast, RPR is reduced beyond the common core in many lineages, and in some organisms has been cast away and replaced by an entirely proteinaceous enzyme (12, 57).

Bacterial and archaeal RNase Ps followed divergent evolutionary trajectories. In bacteria, with RNase P containing a single protein component, diversification largely occurred through the acquisition of additional RNA expansions. In Archaea, the recruitment of extra proteins was associated with extensive RNA reduction. The additional proteins provided structural robustness, allowing archaeal RNase P to retain only the RNA elements essential for catalysis and critical interactions with tRNA.

### Generality of the Accretion Model.

The results here demonstrate the general utility of the accretion model for understanding the origins and evolution of ancient RNAs. The accretion model offers a phenomenological, layer-based framework in which the evolutionary growth of large RNAs can be rationalized and visualized. It establishes a set of rules and constraints inferred from the three-dimensional structures of extant molecules, providing a plausible path from primordial fragments to the mature complexes observed in modern biology. Multiple in vitro selection experiments show that evolutionary paths that lead to novel core structures are rare for functional RNAs (59–62) and that elaboration upon conserved RNA structures readily leads to enhanced fitness (62, 63). It therefore appears that a tendency to evolve through accretion, i.e., growth through elaboration that does not significantly alter preexisting core structures, is intrinsic to RNA evolution.

The accretion model captures the logic of stepwise coevolutionary growth of RNAs in three dimensions, disfavoring fusion of large preexisting RNA modules (13). Our model is agnostic to specific chemical mechanisms of RNA expansion. Initial AES association can be noncovalent (13, 64) or covalent, and insertion can be catalyzed by ribozymes or occur nonenzymatically (65–67). Accretion provides a general sampling mechanism where new elements are either selected for function (folding and resistance to degradation, metal binding, and/or catalytic activity) or accreted through nonadaptive processes (68, 69), as described for rRNA (70, 71) and RNase P evolution (11).

### The Significance of Secondary and Tertiary Interactions.

Primary, secondary, and tertiary structures of RNA are objective elements of molecular organization. The primary structure is the linear nucleotide sequence. The secondary structure is composed of regions of base pairing. The tertiary structure consists of a distinct subset of molecular interactions that form post hoc to secondary structure, both in folding pathways and over evolutionary time. During RNA folding, secondary interactions form first, followed by tertiary interactions (72, 73). The same rules govern RNA evolution: Secondary interactions are incorporated through the accretion of AESs, with tertiary interactions between AESs emerging subsequently (6, 7). Tertiary structure is necessarily dependent on secondary structure. The converse is not true. This hierarchy reflects a self-consistent and predictive model of structure, stability, and evolution of RNA structure.

Recognition of P4 as a secondary structure element is based in part on experiments that indicate that P4 forms before P2 during RPR folding (29). Additional support for P2 as a tertiary element comes from examination of archaeal (Fig. 3) and mitochondrial (mt)–RNase P (74) RPRs, some of which lack P2 (e.g., some RPRs within *Nanopusillaceae* and mt-RNase P RPRs from ascomycete fungi, *SI Appendix*, Fig. S11). By contrast, P4 is universally conserved. The presence of P4 in the absence of P2 is consistent with the independence of secondary from tertiary structure. P2 is not a structurally essential component of RNase P. Indeed, Westhof and coworkers argued previously for the P4 secondary/P2 tertiary assignment (Fig. 2*B*) (30).

In the traditional secondary model of RPR (18, 28, 41, 75) helix P2 is represented as a secondary element and helix P4 as a tertiary element (pseudoknot) (Fig. 2*A*) (76); or, in the case of wire schematics (75), P2 and P4 are represented equally. In those cases, P4 is still frequently referred to as a tertiary element. Computational approaches to pseudoknot resolution were unable to unambiguously identify which element is the tertiary element in RPR (31).

We used both the folding-based and the traditional secondary structural models separately to model the evolution of RPR by accretion (Fig. 4 and *SI Appendix*, Fig. S1 and *Text*). An evolutionary trajectory based on the traditional P2 secondary/P4 tertiary structure explodes into many poorly restrained possibilities and an entangled region (*SI Appendix*, Fig. S1*A*). By contrast, an evolutionary trajectory based on the folding-based P4 secondary/P2 tertiary structure converges into a well-defined evolutionary trajectory. Thus, correct partitioning of RNA secondary and tertiary elements is necessary to determine correct evolutionary trajectories.

## Conclusions

RNase P has much in common with the ribosome. Both are multiple turnover ribozymes, both perform functions required for translation, both are RNA–protein complexes that evolved through accretion, and they share common structural motifs (45, 77). The data support a model of coevolution of RPR, tRNA, and rRNA reflecting shared functional constraints and evolutionary pressures in a common milieu. By sampling RPR sequences and structures across the phylogeny and partitioning the structures into the accretionary elements, we can estimate states of RNase P prior to LUCA, at LUCA, and after LUCA. The data support a model in which stepwise evolution of RPR was followed by lineage-specific deletions and accretions post-LUCA. The accretion model reveals how AESs evolved to create structures that recognize pre-tRNA and catalyze the cleavage of the 5′ leader.

The model presented here supports the accretion process as an intrinsic feature of evolution of structural RNA molecules at the dawn of life. Application of the accretion model requires correct partitioning into secondary and tertiary elements. The model provides blueprints for future experimental work for evaluating changes in structure, activity, and specificity along specific, predicted evolutionary paths. Ancestral elements of RNase P proposed in the current study should be probed for folding, assembly, and catalytic activity with pre-tRNAs and alternative substrates (78, 79).

## Methods

We used a combination of structural analysis and visual inspection, phylogenetic, and statistical approaches to investigate the structure and evolution of RNase P and its behaviors across the tree of life. We incorporated data from RNA and protein components of bacterial, archaeal, and eukaryotic RNase P. As described in detail in the *SI Appendix* *Text*, our approaches incorporated the accretion model and analysis of crystal and cryo-EM structures; multiple sequence alignments and tree construction; mapping of structural elements onto phylogenies; statistics and clustering of evolutionary trends; generation, revision and analysis of secondary structural models, and searches for homologs of protein components. We also performed ancestral reconstructions of RNase P at LUCA.

## Supplementary Material

Appendix 01 (PDF)

## Data Availability

Sequences, alignments, genomic and phylogenetic data, secondary structures, and 3D representations of evolutionary models associated with this manuscript have been deposited in the FigShare repository (https://doi.org/10.6084/m9.figshare.29545817) (27).
