# Supplementary material for: Coevolution of RNase P and the ribosome
Source: Proc Natl Acad Sci U S A. 2026 Mar 2;123(10):e2518495123. doi: 10.1073/pnas.2518495123 (PMC12974513; doi:10.1073/pnas.2518495123)
Supplement: Supplementary file 1 — Appendix 01 (PDF) [file pnas.2518495123.sapp.pdf]

Supplementary Information Appendix for:

Co-evolution of RNase P and the Ribosome

Anton S. Petrov[a,b], Claudia Alvarez-Carreno[a,c], Loren Dean Williams[a,b], Mark A. Ditzler[d]

[a]NASA Center for the Origins of Life, Georgia Institute of Technology, Atlanta, GA, USA
[b]School of Chemistry and Biochemistry Georgia Institute of Technology, 315 Ferst Drive NW, Atlanta, GA, USA
[c]Department of Structural and Molecular Biology, University College London, London, United Kingdom
[d]Center for the Emergence of Life, NASA Ames Research Center, Moffett Field, California, USA

SI Information

SI Methods
SI Text
Figures
Figure S1. 2D and 3D representations of two versions of the accretion model (with P2 and P4 denoted as pseudoknot)
Figure S2. Phylogenetic distribution of elements across archaeal and bacterial species.
Figure S3. Detailed representations for 2D structures used in the current study.
Figure S4. 2D structures from Fig. S3 with mapped A-minor tertiary interactions and conserved regions.
Figure S5. Contacts of RNase P -tRNA interactions.
Figure S6. Structural and functional similarities are observed between the Catalytic Domain of RNase P, and the Peptidyl Transfer Center (PTC) of the LSU rRNA.
Figure S7. LUCA and post-LUCA architectural diversification.
Figure S8. Distribution of RNase P length among archaeal and bacterial species.
Figure S9. Distribution of P18/P18.0 within AES2 in bacterial, archaeal and eukaryotic RNase P at the levels of sequence and 3D structure.
Figure S10. Summary of protein homology search performed for archaeal RPPs.
Figure S11. Size distribution of variable elements P12.1 and P19.
Figure S12. Revised secondary structures of reduced RNPs
Figure S13. Relationship between AESs and Phases mapped onto secondary structure.

Figshare Files https://doi.org/10.6084/m9.figshare.29545817:
File S1. Sequences & MSA of bacterial RPR
File S2. Sequences & MSA of archaeal RPR
File S3. Genome annotations, presence or absence of ancestral expansion segments within RPR
File S4. Supplementary Data for mapping phylogeny of RPR onto evolutionary tree (for Figure 3 of the Main Document)
File S5. PyMol script and 3D structures of RNase P from different three domains of life depicting the accretion model of RPR evolution.
File S6. The revised 2D structures of various archaeal and bacterial RPR (svg, json).zip

Tables
Table S1. Experimental structures to build the accretion model of RNase P evolution.
Table S2. Correspondence between helical elements of RPR and ESs/AESs
Table S3. Accretion model data (Ancestral Expansion Segments, Ranges, Structures)
Table S4. Accretion model data (Expansion Segments, Ranges, Structures)
Table S5. List of the secondary structures remodeled
Table S6. Summary of A-minor interactions

Methods

Sequences, MSA, and phylogeny

Genomic sequences were obtained using accession numbers from a curated dataset of genomic assemblies compiled by Moody et al. (1). This comprehensive dataset contains 349 archaea and 350 bacteria and encompasses all major archaeal and bacterial lineages. It includes 71 metagenome-derived bacterial assemblies and 198 metagenome-derived archaeal assemblies. Information on genomic/metagenomic abundance as well as completeness of RNase P genes and percentage of metagenome contamination is given in File S3.

RPR sequences were retrieved using the Infernal package (2) with four distinct covariance models (3) of RNase P RNA from the Rfam database: RF00010 (Bacterial RNase P class A), RF00011 (Bacterial RNase P class B), RF00373 (Archaeal RNase P class A), and RF02357 (Archaeal RNase P class T)(4, 5). All hits with E-values below 0.01 were retained (302 archaeal and 339 bacterial assemblies) and inspected to identify helices P2, P3, P5, P5.1, P8, P12, P14, P15, P16, P6, P18.0, P18, and P19 (FigShare File S3). In most cases, alignments to the secondary structures generated by Infernal were used to define helices; however, in some cases poor alignment to the covariance models required partial manual identification of secondary structure elements. When necessary, manual identification was made by visual inspection of potential base-pairing patterns within these helices to verify Watson-Crick and wobble base pair compatibility, and in some cases local secondary structure prediction was performed with RNAfold (6). Some sequences also required comparison with closely related and/or experimentally validated secondary structures to ensure proper identification of structural elements.

RPR elements were mapped onto a maximum-likelihood phylogenetic tree of Archaea and Bacteria (LG + C60 + G4 + F model) adapted from Moody (1). The tree was inferred from a concatenated alignment of 27 vertically evolving genes. A detailed description of the phylogenetic inference is provided in the original source (1). A visualization of the phylogenetic distribution of RPR elements was generated in iTOL (7). One archaeal taxon from the original dataset (B21_G17) could not be mapped to a GenBank assembly and was excluded from subsequent analyses.

The Infernal package (2) and covariance models were also used to generate separate multiple sequence alignments for the archaeal and bacterial RPRs. The resulting alignments are provided (FigShare File S1 and File S2).

2D structures

Revised RPR secondary structures in the current study and the mapping of various structural data were performed using Exornata (8), a web-based tool available at https://exornata.chemistry.gatech.edu. Exornata is an expanded, browser-based version of the original XRNA software developed by Harry Noller (available at http://rna.ucsc.edu/rnacenter/xrna/xrna.html). The initial set of archaeal and bacterial 2D models was incorporated into the local version of R2DT (9, 10) and used for automated generation of the remaining templates.

Applying the secondary scheme in which P4 is a secondary element and P2 is a tertiary element, we generated revised secondary layouts for: (i) RPRs from three-dimensional (3D) crystal and cryo-EM structures analyzed in the current study, and (ii) RPRs previously described by Brown (11). For the subset RNase Ps of known 3D structure, base-pairs were extracted from 3D structures using DSSR (12).

We modeled two reduced RPRs that exhibit substantial deletions within the catalytic domain. These structures are from the archaeon Candidatus *Nanobsidianus stetteri* and from the mitochondrion of the fungus *Saccharomyces Cerevisiae* reported previously by Lang and coworkers (13), for which the functional activity has been demonstrated by Martin and co-workers (14). The reduced RPR of *Nanobsidianus stetteri* has been previously described in several members of Nanoarchaeales (Nanopusillaceae) (15, 16), for which three protein components of RNase P have also been identified, suggesting the activity of the RNase P. Both of these RPRs lack both helices P3 and P2. These reduced structures demonstrate the evolutionary plasticity of RPR; pseudoknot P2 is an important but not essential feature for RNase P function.

3D structures

Three-dimensional structures of RNase P complexes encompassing representatives from all three domains of life (Table S1) were obtained from the Protein Data Bank (PDB) (17). The dataset included bacterial RNase P structures from *Thermotoga maritima* (PDB IDs: 3Q1R, 3Q1Q) (18), *Thermus thermophilus* (1U9S) (19), *Bacillus subtilis* (1NBS) (20), and *Geobacillus stearothermophilus* (2A64, 3DHS) (21, 22), archaeal RNase P structure from *Methanocaldococcus jannaschii* (6K0B, 6K0A) (23), and eukaryotic RNase P structures from *Homo sapiens* (6AHU, 6AHR) (24) and *Saccharomyces cerevisiae* (6AH3, 6AGB) (25).

Detection and visualization of expansion segments (ESs) and ancestral expansion segments (AESs) was performed within the program PyMOL (26). Global structural superimpositions of bacterial, archaeal and eukaryotic structures were performed using the built-in CEalign (27) of PyMOL. ESs and AESs were identified by rules given below. RNase P RNA structures were partitioned into ES/AES objects in PyMOL, that were colored individually and organized according to the temporal accretion. The resulting PyMOL script is given as Figshare File S5.

Remote Protein Homology

To identify remote homologs of archaeal ribonuclease P protein components Rpp21, Rpp29, Rpp30, Rpp38/uL8, and Pop5, and the bacterial ribonuclease P protein component rnpA/C5, profile hidden Markov models (pHMMs) were constructed with HMMER3 (28) from multiple sequence alignments generated using MAFFT v7.505 (29). The resulting pHMMs were searched against genome assemblies from 349 archaeal and 350 bacterial species, following the sampling of Moody (1). Hits passing the threshold of E-value < 1×10-2 were retrieved and clustered with CLANS (30). Sequences were re-aligned by cluster and compared pairwise with hhalign from HH-suite3 (31).

Accretion

ESs/AESs are RNA fragments excised from 3D structures of RNA molecules as determined by coaxial continuity of stacking interactions, punctuated by insertion fingerprints. The approach is described in detail by Petrov and coworkers (32, 33).

Identifying Lineage-Specific Expansion Segments

Lineage-Specific ESs were identified by comparison of the common core rRNA and RPR to extant rRNAs and RPRs. Upon an RNA expansion, new branch RNA is inserted into old trunk RNA (32, 33). Insertion fingerprints are evident upon inspection of the structures in three-dimensions. On either side of the branch site, the sugar and phosphate moieties of the trunk helix are in proximity to each other, such that if a branch segment is excised, the trunk could be resealed with a minimal perturbation of the nucleotides near the insertion site. We observe that helices of trunk segments show minimal distortion at branch sites. Stacking is continuous within the trunk and generally within the branch, but not generally between the trunk and the branch. The helical axis of branch segments deviates acutely from that of the trunk helix. The characterization of trunk versus branch is in some cases dependent on external indicators of relative order of addition.

Identifying Ancestral Expansion Segments

Insertion fingerprints, with structural characteristics similar to those described above for lineage specific expansions, are observed throughout the RPR and ribosomal common cores. These ancestral insertion fingerprints allow us to infer AESs (Table S3), which appear to build up the common core. Continuity of the sugar-phosphate backbone, stacking interactions, and local helical axes were used to help define AESs. Our model is an under-sampling of the actual expansion events. In our model, the majority of the AESs were added at their present size without elongation. In reality, many AESs increased in length over time through helical elongation. However, helical elongation does not leave fingerprints and so is not included in the model.

Accretion rules for RPR

The following rules were used to identify expansion segments and determine their chronology for the accretion model of RPR:

1) **Identifying expansion segments:** expansion segments were identified by inspection of structures in three-dimensions. The following features were used to identify expansion segments:
    a. The 5′ and 3′ ends of the segment are sufficiently close together in the 3D structure that if the expansion segment were removed, backbone continuity could be reestablished through minimal structural adjustment of the nucleotides immediately flanking the expansion segment.
    b. Any secondary structure element present within a given segment must be complete, i.e., the two sides of a helix within the secondary structure cannot be split between two different AESs.

c. AESs do not contain any internal tertiary contacts; tertiary interactions only occur between AESs.

Backbone continuity within an expansion segment can only be broken by the insertion of another AES that satisfies the above criteria.

2) **Chronology of expansion:** AESs represent survivors of much broader sampling of ancestral RNA fragments that were incorporated over time into larger RNA complexes. The numbering scheme reflects their order of incorporation into a specific RNA molecule, not their relative age. A model of this temporal ordering of expansion segments was based on topological and structural features of RPR using the following constraints.
   a. Topological connectivity is maintained throughout the accretion process; accretion is treated as a continuous process without any segment rearrangements or circular permutations.
   b. In an integrated system, independent structural elements are inferred to be older than dependent elements. Pairs of such elements are exemplified by A-minor motifs (1), in which an A-minor donor is structurally and functionally dependent on its A-minor acceptor (2, 3). Consequently, an AES that functions as an A-minor acceptor is ranked as evolutionarily older than an AES that functions as the corresponding donor. A-minor interactions were identified by examining all adenosines not engaged in canonical base pairs and determining which had nucleobases positioned to form hydrogen bonds with the minor groove of another helix. A conservative hydrogen bond distance cut-off of 3.2 Å was applied, corresponding to the separation distance typically observed between hydrogen bond donors and acceptors.
   c. The two components of an RNA pseudoknot can be distinguished as either the *invading* or the *invaded segment*. Strand invasion is a molecular process in which a single-stranded region of RNA invades a double-stranded region of RNA by pairing with one of the duplex strands, displacing the other strand. In RNA the invading strand typically originates from a loop or single-stranded region

The chronology of late expansions is poorly defined, as late expansions at the surface of RNA may be added to different regions independently from each other.

Identifying post-LUCA insertions and deletions

As part of this work, we wanted to understand the state of RNase P at LUCA. In this regard we used the broadest available data set that samples species within bacterial and archaeal phyla. Using this data set, we mapped helical elements of RNase P and obtained their distribution across phylogeny. If a helical element is present in deeply rooted clades within both bacteria and archaea, we assume that element to be part of RPR at LUCA. This approach enabled us to infer a parsimonious state of RNase P at LUCA, which contains P1, P2, P3, P4, P5, P6, P7, P8, P9, P10, P11, P12, P15, P16, P17, and P19. Significantly, the data support a model in which P16, P17, and P19 were present within RPR at LUCA. Using this definition of RPR at LUCA, we assign insertions and deletions within individual lineages.

P12.1 and P19 correlation studies

To investigate the variation in the P12.1/P12.2 and P19 across the archaeal domain, we conducted a clustering analysis based on correlated growth patterns in these regions (Fig. S11A). We extracted the variable-length segments of P12.1/P12.2, located between the strands of helix P12 (alignment positions 496–913), and P19, located between strands P2 (3′) and P4 (3′) (alignment positions 1243-1365), from the archaeal RNase P alignment (FigShare File S2). Alignment gaps were removed, and sequence lengths of each segment were computed.

We applied K-means clustering to identify natural groupings of species based on segment length profiles. The optimal number of clusters was determined using the elbow method, which detects the point at which additional clusters yield minimal improvements in within-cluster sum of squares (inertia). The elbow point (Fig. S11B) indicated an optimal value of k = 3. Accordingly, K-means clustering with k = 3 was used to partition the dataset into three distinct groups (Fig. S11C) based on the P12.1/P12.2 and P19 length distributions.

To visualize evolutionary trends, segment length values were mapped onto a phylogenetic tree (Fig. S11A) of archaeal species constructed using iTOL (7)). Each expansion segment was represented as a bar aligned with the corresponding species to reflect segment size and distribution across the phylogeny.

SI Text

Our revision of the secondary structure of RNase P

The discovery of RNase P (34, 35) led to efforts to predict the secondary structure of its RNA (RPR) (36). The original secondary structure of RPR (Fig. 2A, Main Text) proposed by Pace (37) depicts a core of RPR with 15 helical elements connected by junctions. P2 was identified as secondary helix and P4 was identified as a tertiary helix (38, 39), which was denoted as a pseudoknot. Subsequent studies questioned the characterization of P4 as a tertiary element and suggested reassigning the pseudoknot to P2. Specifically, Williamson showed that i) P4 is longer than P2; ii) P2 forms after P4 in kinetic studies (40); iii) P4 is buried deep in the structure while P2 was exposed on the surface (41). Based on this information, Westhof et al. proposed a secondary model of RNase P, in which P4 is a secondary element and P2 is a tertiary element (42). As of today, even the advanced sequence-based methods, while providing a strong support for detection of both helical regions P2 and P4, demonstrate a weaker co-variation within P4 compared to P2 due to high conservation of the sequence within the catalytic site (43). Various computational algorithms for pseudoknot removal resulted in ambiguous characterization of pseudoknots within RNase P: one group of methods favored P2, the others supported P4 (44).

Crystal structures of RNase P from species representing the three domains of life revealed conserved and divergent features of RNase P, including atomic details on base-pairing, and stacking arrangements with helical elements (21, 23, 45). The 3D structures also confirmed the presence of a pseudoknot. Yet the assignment of the pseudoknots (P4 as tertiary interaction versus P2 as tertiary interaction) was not reconsidered. As of today, the most broadly used secondary structural layout of RPR depicts P4 tertiary interaction (4, 5, 46). Secondary structures of RPRs have also been depicted as wire diagrams, in which

secondary and tertiary interactions are not differentiated, and P2 and P4 helices are given equal status (45, 47).

Incompatibility of the accretion model with the traditional secondary structure of RNase P

The assignment of AES elements based on the traditional RPR secondary structure is shown in Fig. S1. Using this secondary structure as a blueprint for RNase P evolution, the catalytic core would be expected to form from the union of helices P1–P3, followed by the incorporation of P5–P15, and the formation of the tertiary pseudoknot P4. However, this partitioning reveals several critical issues that conflict with the core principles of the accretion model.

First, if P4 is treated as a tertiary interaction, the expansions formed by helices P1 and P4 (along with P5 and P15) create a topological entanglement (Fig. S1A) that violates the assumption of structural continuity by accretion.

Second, if P2 is considered a secondary structure element, then along with P3, it would form an expansion segment inserted into both P1 and P4. This implies that the formation of the P1–P5 region required a coordinated assembly—likely involving multiple RNA splicing or recombination events—rather than a sequential stepwise accretion.

Third, such violations of continuity introduce uncertainty in the chronological ordering of RNA expansion elements. This model leads to numerous, mutually exclusive scenarios for the origin of RNase P, none of which would support its emergence through a gradual accretion mechanism.

Why pseudoknots matter

The P2 versus P4 pseudoknot consideration extends beyond static structural biology, it is also a question of a) RNA folding dynamics, b) RNA evolution, and c) structure-function relationships. The distinction between P2 and P4 is essential to understanding these processes.

The 3D structures used to formulate the accretion model are static objects, which do not directly reveal their folding pathways. The P2 versus P4 issue is not strictly structural as it also requires an understanding of the RNA folding and evolutionary pathways. RNA folding and evolution are hierarchical.

Thus, the distinction between secondary and tertiary interactions is also intrinsic to an evolutionary model. We attempted to develop an evolutionary model in which the P2 was designated as secondary interaction and P4 was a tertiary interaction. That model failed at many levels. We found that the only viable evolutionary model requires P2 as a tertiary interaction and P4 as is secondary interaction.

Revised secondary structures

Revised secondary structures for several model species (listed in Table S5) that represent various structural types and sub-types of RNase P as defined in ref. (11). The revised representation explicitly depicts P4 as a secondary helical element within RNase P secondary structure coaxially stacked with P1 and P5. and treats P2 as a tertiary pseudoknot.

Additional lineage-specific deletions

In addition to the loss of P6 (AES2/AES4), P13 and P14 (ES5.3) along the Bacilli-Mollicute lineage described in the main text, these elements are also lost within much of candidate phyla radiation (CPR). Within CPR, the absence of these elements from RPR often coincides with the presence of ES 1.1 and ES 2.2.1 (as observed along the Bacilli-Mollicute lineage); however, several RPRs within CPR that lack P6 and/or P13 and P14 do not have ES 1.1 or ES 2.2.1. In those cases, it is unclear what, if any, element within RPR compensates for the loss of these structural elements.

Another common mode of sequence deletion to the ancestral core is the loss of P19 (AES2), which is sporadically lost across all three domains of life. When present, P19 coaxially stacks on P2, and P2 provides a large fraction of the binding site for the single RNase P protein in Bacteria. P19 is also positioned near the 5′ leader sequence of the pre-tRNA and may have played a role in leader sequence recognition before the addition of the bacterial RNase P protein, which is responsible for recognition of the 5′ leader sequence in modern RNase P. The addition of the protein may have allowed for the deletion of P19 by providing alternative structural stabilization to P2 and by taking on the role of 5′ leader recognition. We also note that P19 is generally excluded from previously proposed ancestral forms of RNase P, which is present in our model of LUCA. This exclusion of P19 from prior models of LUCA likely reflects the absence of P19 in *E. coli* RNase P, which is widely used as an exemplar of the ancestral form.

Across Archaea, P2 (AES2/AES3) is also sporadically lost (Fig S11A). The dispensability of P2 is consistent with its identity as a pseudoknot, and its role in stabilizing the structure of the catalytic domain may now be dispensable with the addition of multiple proteins to RNase P.

Analysis of RNase P proteins

Archaeal RNase P contains five proteins: Rpp21, Rpp29, Rpp30, Rpp38, and Pop5. Our inspection of archaeal RNase P reveals that three proteins—Pop5, Rpp21, and Rpp29—directly interact with RPR (Fig. S7D). Pop5 binds to AES2 and the P2 pseudoknot near the catalytic site and interacts with the 5' leader. Rpp21 binds to AES1 and Rpp29 binds to AES6; both of these proteins interact with the elbow of the tRNA, and by forming contacts with each other, they essentially form a bridge between the catalytic and specificity domains. Protein Rpp30 binds to Pop5 and Rpp29, stabilizing the junction between AES1 and AES2 and forming an additional contact with the acceptor stem of tRNA, thus enhancing the stability of the RNase P-tRNA complex. Finally, Rpp38 anchors to Rpp29 and interacts with the K-turn of archaeal/eukaryotic expansion ES5.1 (48), enhancing the stability of the specificity domain.

Sequence analysis reveals that most of the archaeal RNase P proteins have homologous relationships to proteins rooted within the machinery of the central dogma (Fig. S10). Homologs of archaeal Pop5 and its structural bacterial analog C5 that bind near the catalytic site cannot be reliably identified. Bacterial C5 adopts a fold of the C terminal domain of the ribosomal protein uS5. Archaeal Pop5 adopts an RNA-recognition-motif–like fold (RRM) (49, 50). In both cases, sequence similarity is too low to unambiguously establish homology.

Rpp21 shares ancestry with transcription factor S (found in archaea), Rpp29 shares ancestry with the ribosome maturation factor (found within bacteria), and Rpp30 shares ancestry with the universally distributed histidinol phosphatase. Rpp38 may have been incorporated from the archaeal ribosome. Our remote homology analysis using pHMMs also shows evidence of shared ancestry between Rpp38 and the peptide chain release factor (Fig. S10).

Thus, the analysis of protein components within RNase P suggests that it coevolved with other essential components of the translation and transcription machinery that constitute the central dogma, giving rise to life on early Earth.

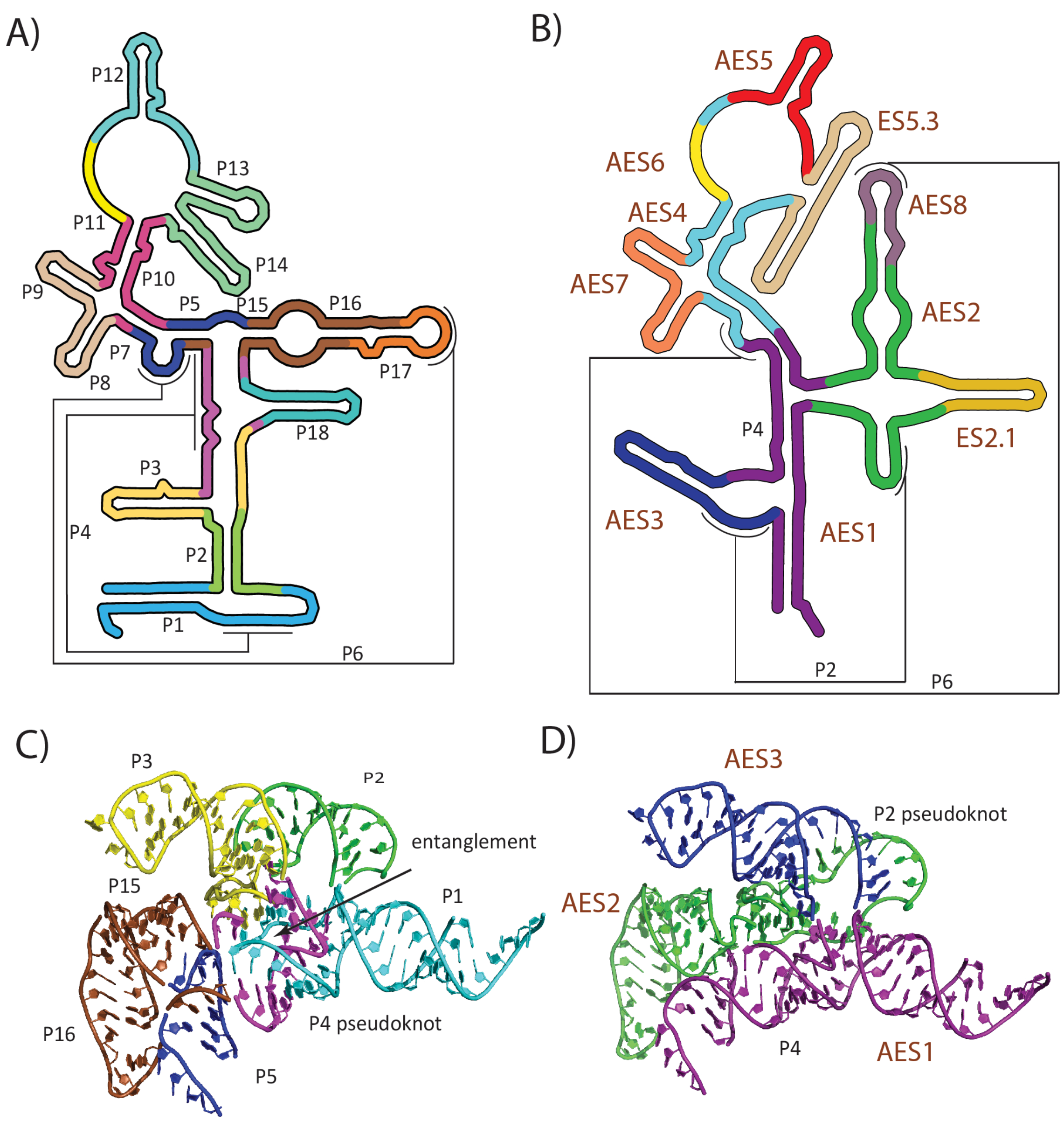

Figure S1. Partitioning of RPR into expansion segments as defined using A) the traditional secondary structure, with P4 as pseudoknot or B) the revised secondary structure, with P2 as pseudoknot. The resulting expansion segments of RPR were mapped onto (A, B) secondary and (C, D) tertiary structures of RPR. Different expansion segments in both models are depicted by distinct colors. The topological entanglement in Fig. S1C appears because the entangled region (P4) is (incorrectly) treated as a tertiary interaction and therefore, the two strands of P4 are partitioned into two separate expansion segments. The entanglement does not appear when both strands of P4 are assigned into the same AES (AES1) in Fig. S1D, where the base pairs across these strands form the secondary interactions. Instead, the P2 (in Fig. S1D) forms tertiary interaction, however the formation of pseudoknot P2 does not create a topological entanglement. This is the only topology that satisfies the continuity of evolutionary accretion and stereochemistry folding.

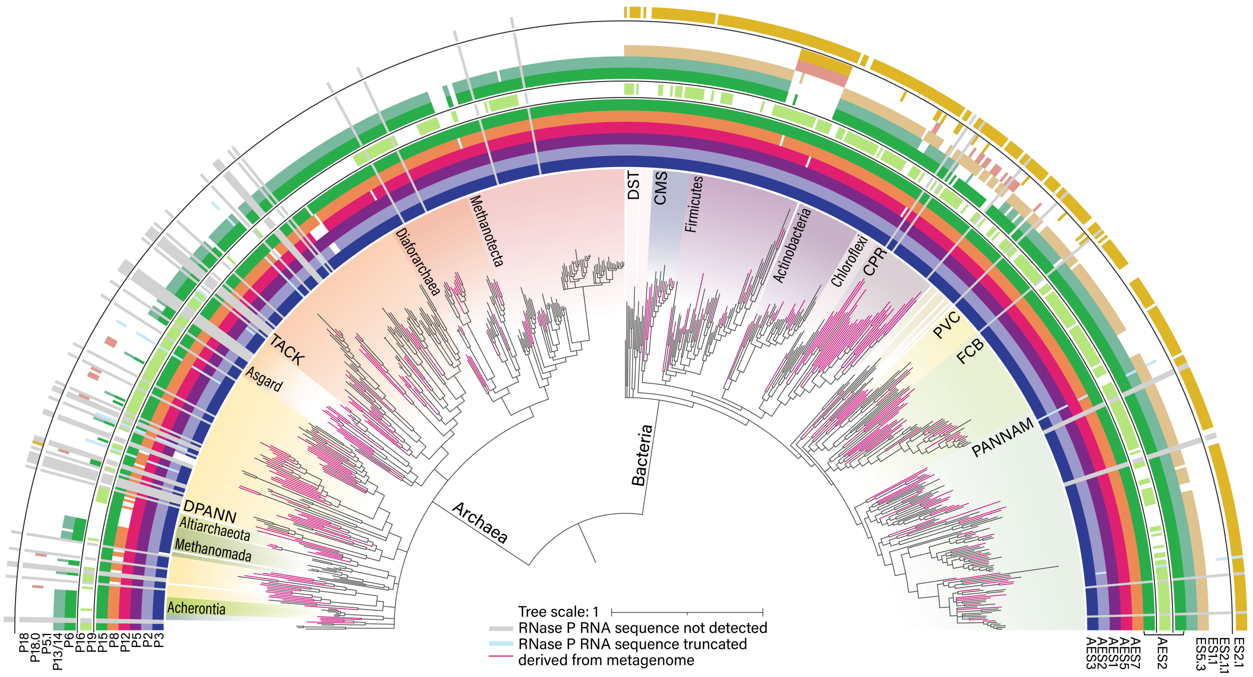

Figure S2. Phylogenetic distribution of RPR secondary structure elements and expansion segments. RPRs were surveyed across 350 bacterial and 349 species archaeal from the tree topology adapted from Moody et al. (1). Species lacking an identifiable RPR are shown with a gray strip. Secondary structure elements from 298 archaeal and 337 bacterial complete RPRs were mapped onto a maximum-likelihood tree of archaeal and bacterial lineages. Incomplete RPR sequences were identified in ten archaeal and two bacterial RPRs (shown in light blue). Pink branches denote metagenome-derived sequences.

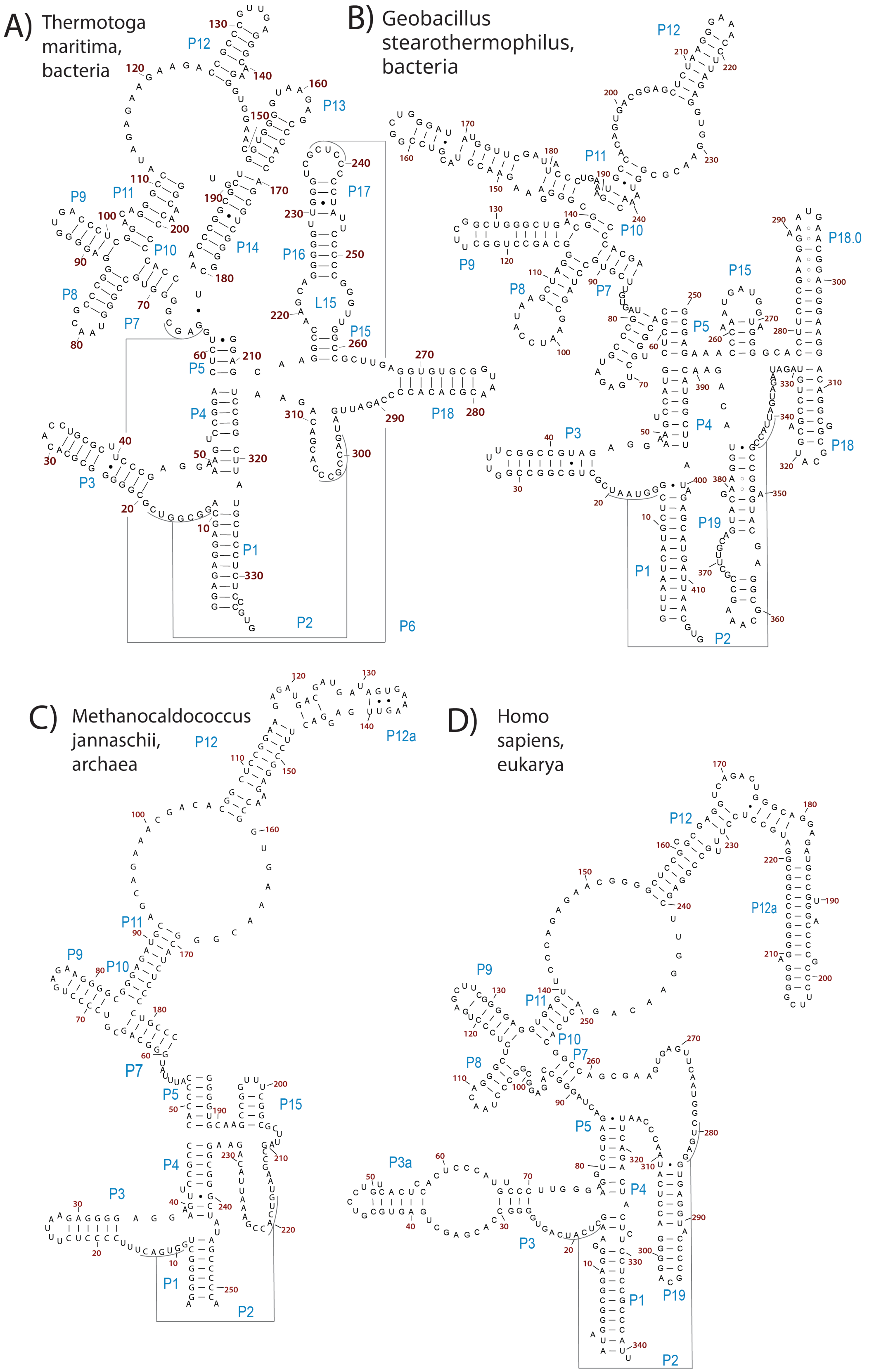

Figure S3. Revised secondary structures of species used in the current study representing the three domains of life. The base-pairs were extracted from the crystal structures listed in Table S1 using DSSR program (12).

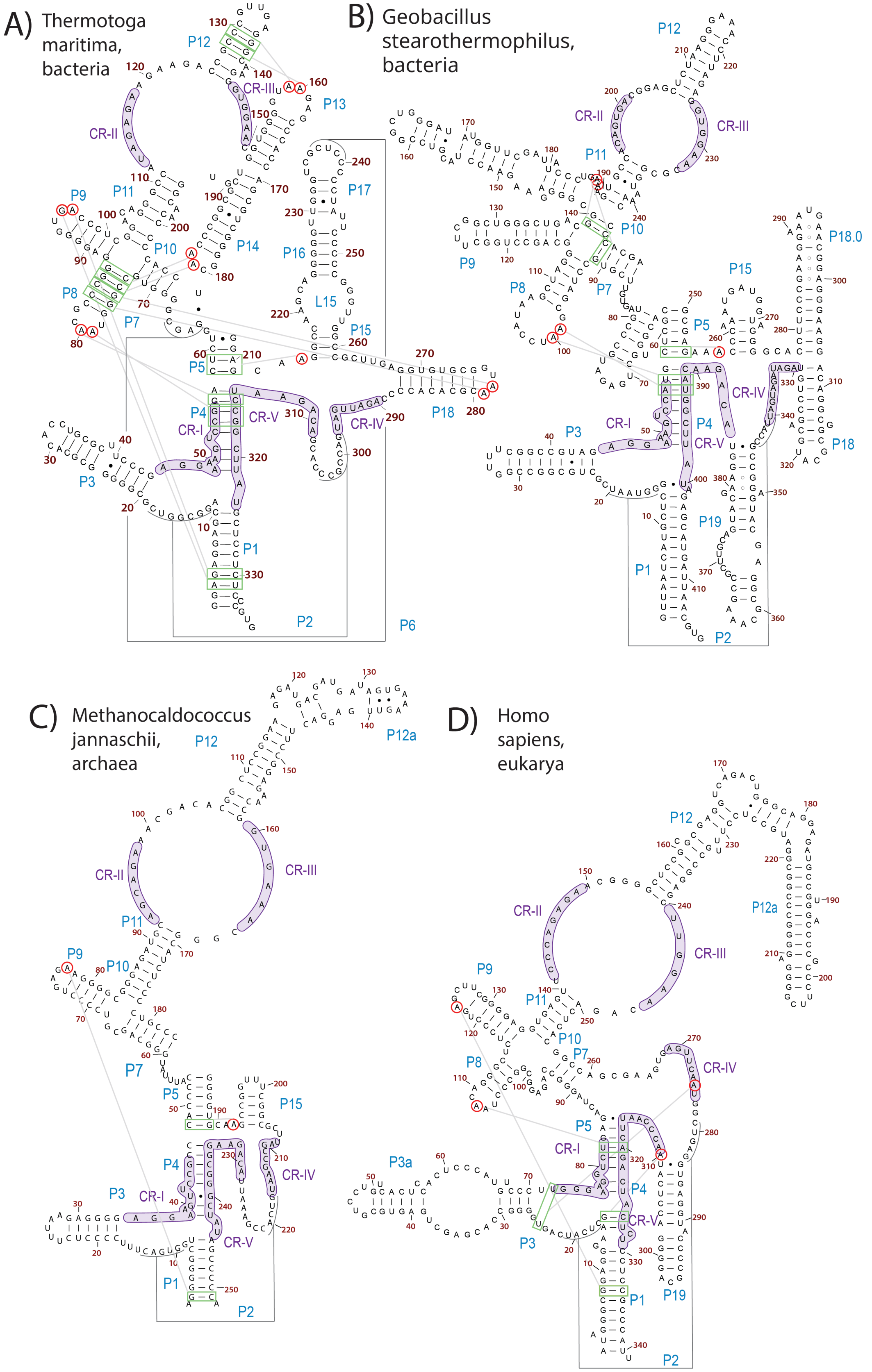

Figure S4. Revised secondary structures of species used in the current study representing the three domains of life. The base-pairs were extracted from the crystal structures listed in Table S1 using DSSR program (12). The conserved regions (CRs) are highlighted in light purple. Tertiary A-minor interactions are marked depicting A donors (red circles) and A-acceptors (green rectangulars).

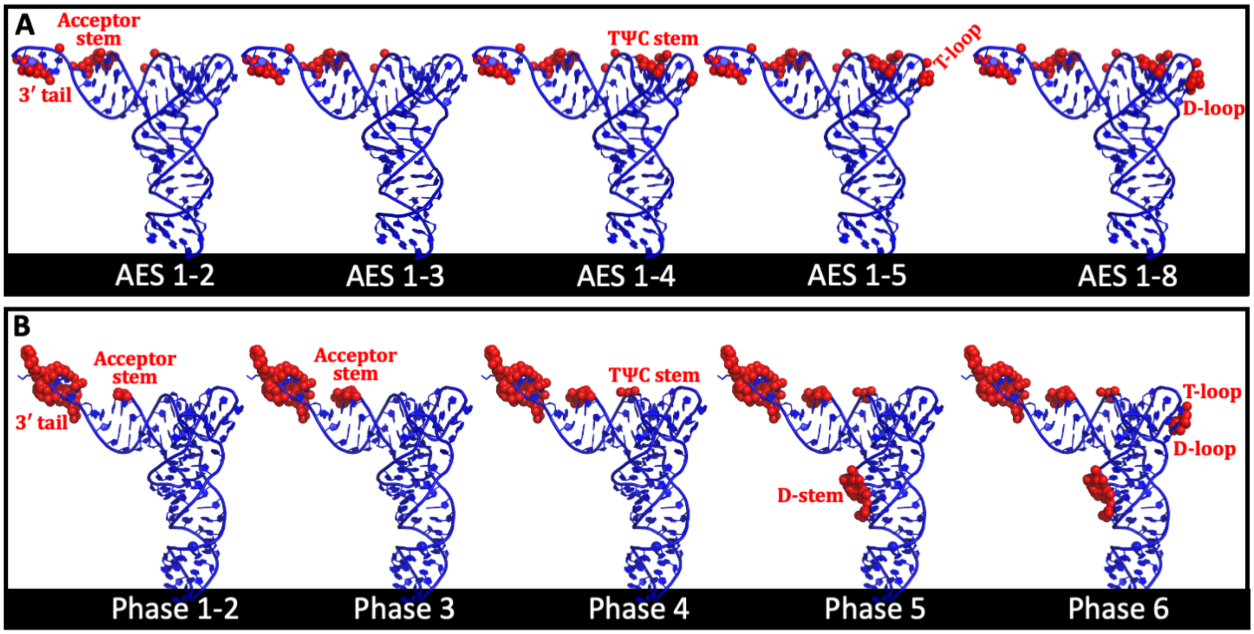

Figure S5. The evolutionary progression of interactions of RNase P with tRNA is broadly similar to the evolutionary progression of interactions of the ribosome with tRNA. (A) The evolution of the RPR:tRNA interface as predicted by the accretion model using the *T. maritima* structure of RNase P. The tRNA atoms in van der Waals contact with the indicated AESs are shown as red spheres. tRNA elements involved in significant new contacts at each step are labeled in red text. (B) The evolution of the rRNA:tRNA interface in the A-site, P-site, and E-site of the large subunit based on AES addition as described in ref. (51). The tRNA atoms in van der Waals contact with the ribosome at the indicated phase of ribosome evolution are shown as red spheres. tRNA elements involved in significant new contacts at each step are labeled in red text.

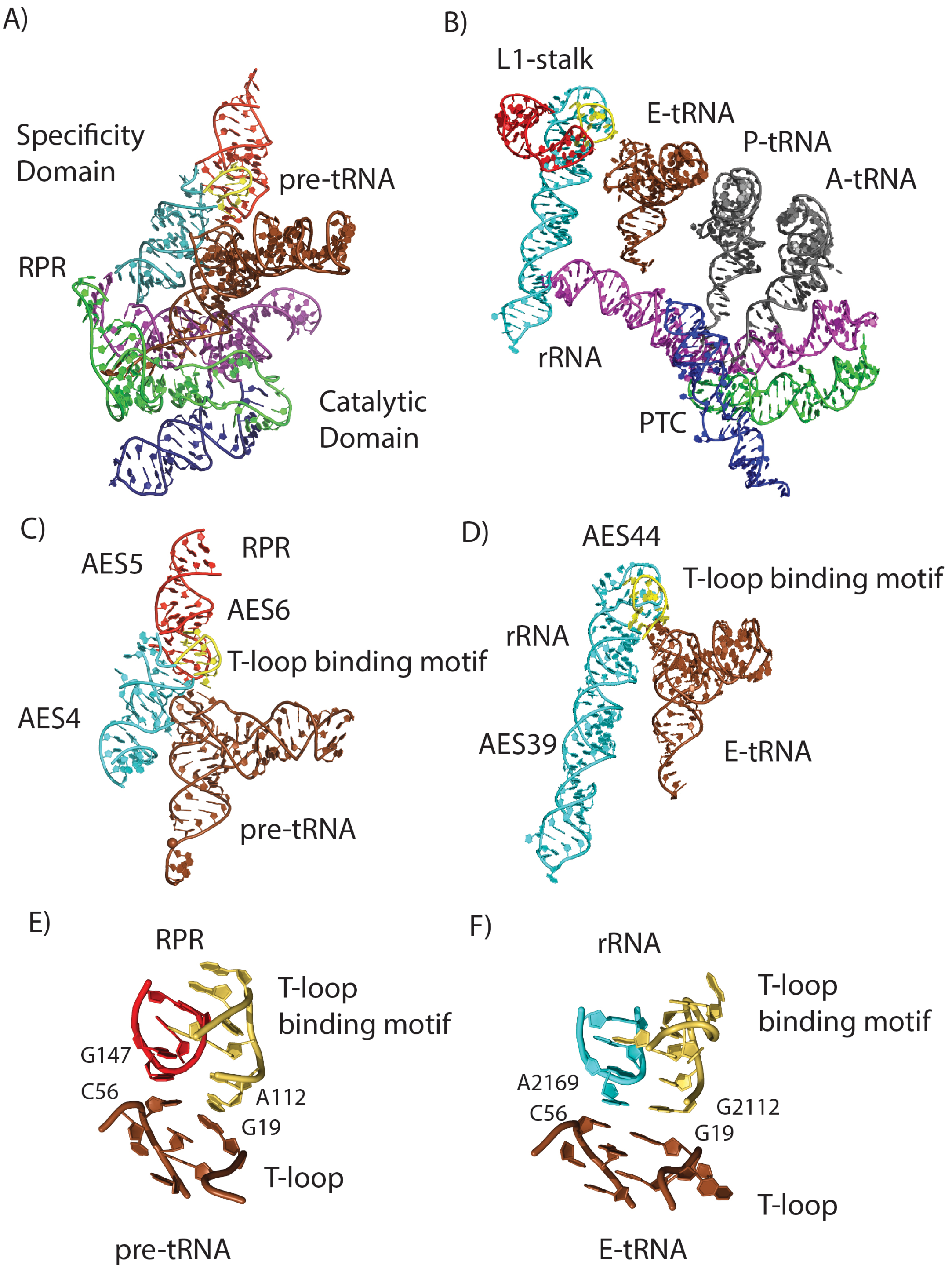

Figure S6. Acute structural and functional similarities are observed between A) the Catalytic Domain of RNase P, and B) the Peptidyl Transfer Center (PTC) of the LSU rRNA. The similarities are particularly striking between the T-loop binding regions of RNR and rRNA. Both of these RNAs interact with the elbow of tRNA. C) RNase P and D) LSU rRNA. Both E) RNase P and D) LSU rRNA structures reveal an identical stacking T-loop binding motif (conserved both in structure and sequence), which further stacks onto tRNA elbow (C56-G19 platform).

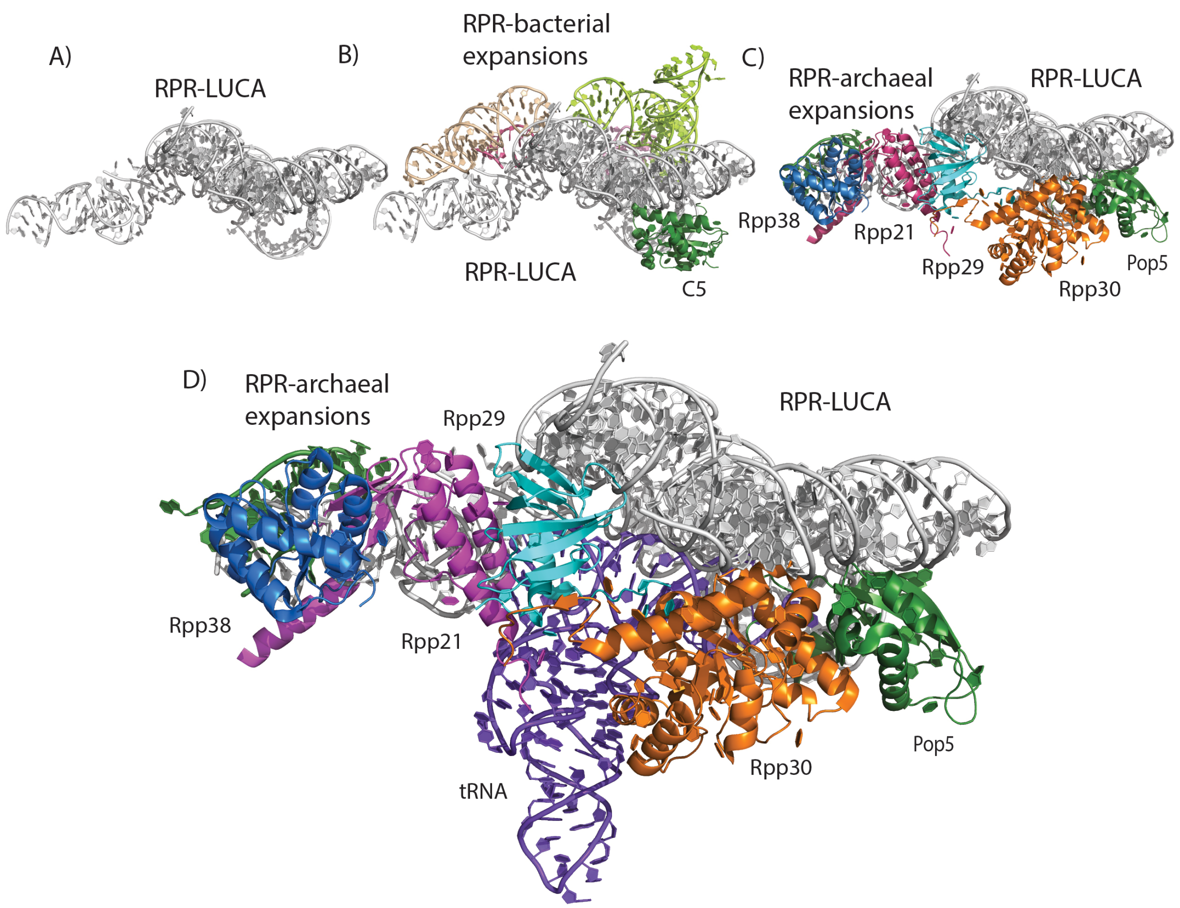

Figure S7. Structural reconstructions of RNase P at A) last universal common ancestor (LUCA), the corresponding secondary structure is provided in figure 5G. B) lineage-specific elements of RNase P within bacterial domain represented by available 3D structures (3Q1R, 2A64, and 1U9S) of bacterial RNase Ps, and C) lineage-specific elements of RNase P within archaeal domain represented by available 3D structure (6K0A) of archaeal RNase Ps. RPR at LUCA is highlighted in gray. Various RNA expansion segments are depicted in various colors. Bacterial protein C5/rnpA is shown in green; archaeal proteins or RNase P are depicted in green (Pop5), orange (Rpp30), cyan (Rpp29), warm pink (Rpp21), and skyblue (Rpp38/eL8). D) Archaeal RNase P within (6K0A) with proteins Pop5, Rpp21, Rpp29, Rpp30, and Rpp38/uL8, colored as in panel C. Four of these proteins (Pop5, Rpp21, Rpp29, and Rpp30) directly interact with tRNA (violet).

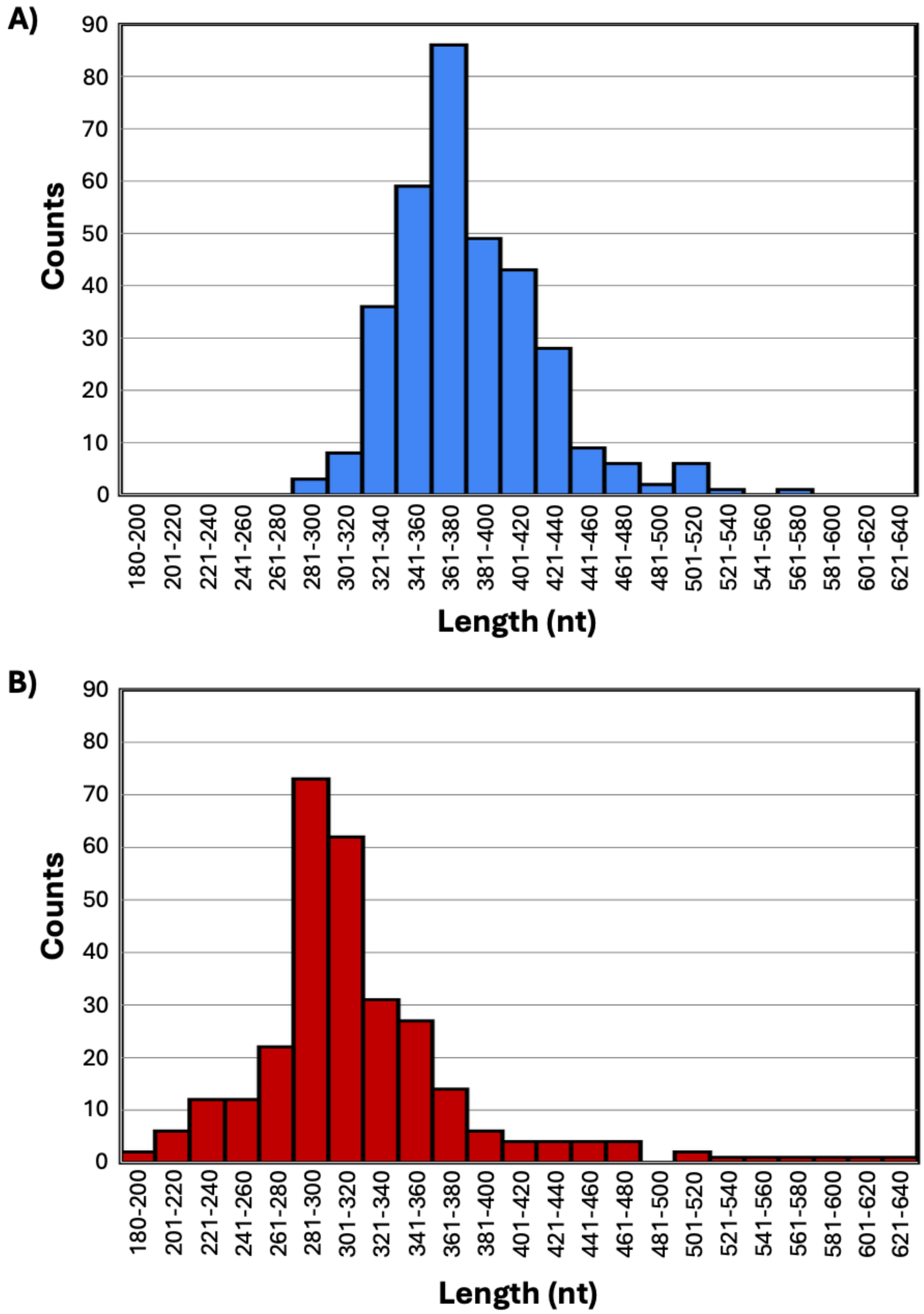

Figure S8. Distribution of the lengths of the detected RPRs in A) Bacteria (mean length: 382 nt; median length: 376 nt) and B) Archaea (mean length: 319 nt; median length: 305 nt) used in the current study. Lengths plotted are approximations based on the length of the sequences recovered from covariance model searches performed using the Infernal software version 1.1.5 (3). The analysis of RPRs was performed using a dataset from a phylogenetic study by Moody et al. (1). Of the 349 archaeal and 350 bacterial genomic assemblies searched, RPR sequences were detected in 302 archaeal and 339 bacterial species. Ten archaeal and two bacterial sequences were identified as incomplete (and were omitted from these distributions). A detailed description of the searched, detected, and incomplete sequences is provided in Figshare File S3. The full sequences are provided in Figshare Files S1 and S2.

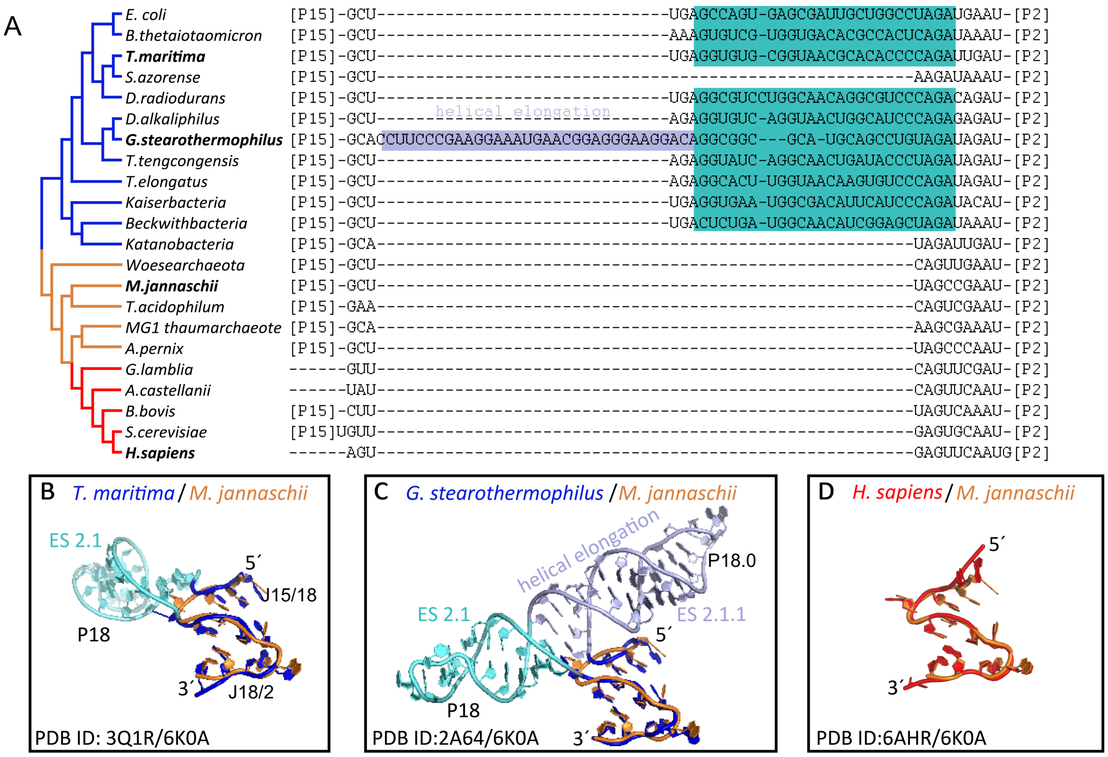

Figure S9. Structure and sequence indicate sites of expansion in RPR. (A) Cladogram and multiple sequence alignment (MSA) of ES 2.1/2.1.1 along with flanking sequences within AES2 are shown for representative species from across the tree of life. The cladogram is based on that of Banfield and coworkers (52), with bacteria blue, archaea orange, and eukarya red. The bacterial expansion segment ES 2.1 is highlighted in the MSA, with the initial insertion in cyan and the subsequent helical extension in light blue. The MSA is colored to match the structures in panels B and C. Species represented in panels B, C, and D are bold in panel A. (B) The structure of ES 2.1 (cyan) from *T. maritima* along with the flanking sequence (dark blue) into which it was inserted. The *T. maritima* structure is superimposed on the corresponding portion of the *M. jannaschii* structure (orange). (C) The structure of ES 2.1 (cyan) and ES 2.1.1 (light blue) from *G. stearothermophilus* along with the flanking sequence (dark blue) into which it was inserted. The *G. stearothermophilus* structure is superimposed on the *M. jannaschii* structure (orange). (D) The structure of the corresponding sequence of *H. sapiens* (red), which lacks ES 2.1, superimposed on the structure of *M. jannaschii* (orange).

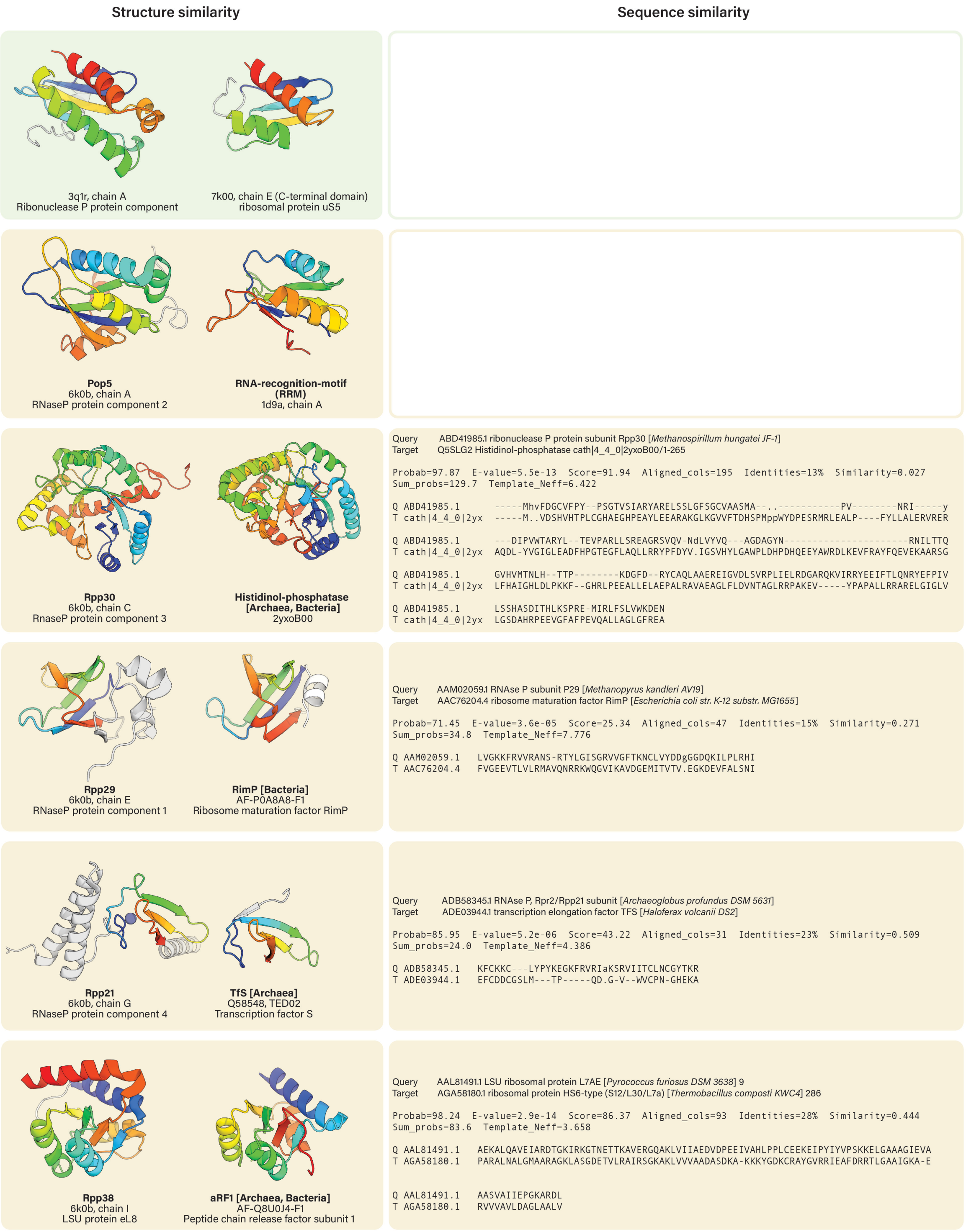

Figure S10. Summary of protein homology search performed for the protein components of RNase P. Cartoon representation RNase P protein structures and their closest homologs are depicted in the left column with structures obtained from experimentally determined structures in the PDB (17), and structure predictions in AlphaFold DB (53) from UniProt entries (54). Structurally similar regions are colored, additional regions are shown in gray. The summary statistics of homology searches (HHalign probability, E-value, score, number of aligned columns, percent of sequence identity, sequence similarity) performed using HMM-based methodology (28) are shown in the right column. HMM-based searches for bacterial C5 and archaeal Pop5 yielded no results, but structure similarities have been reported in the literature (49, 50).

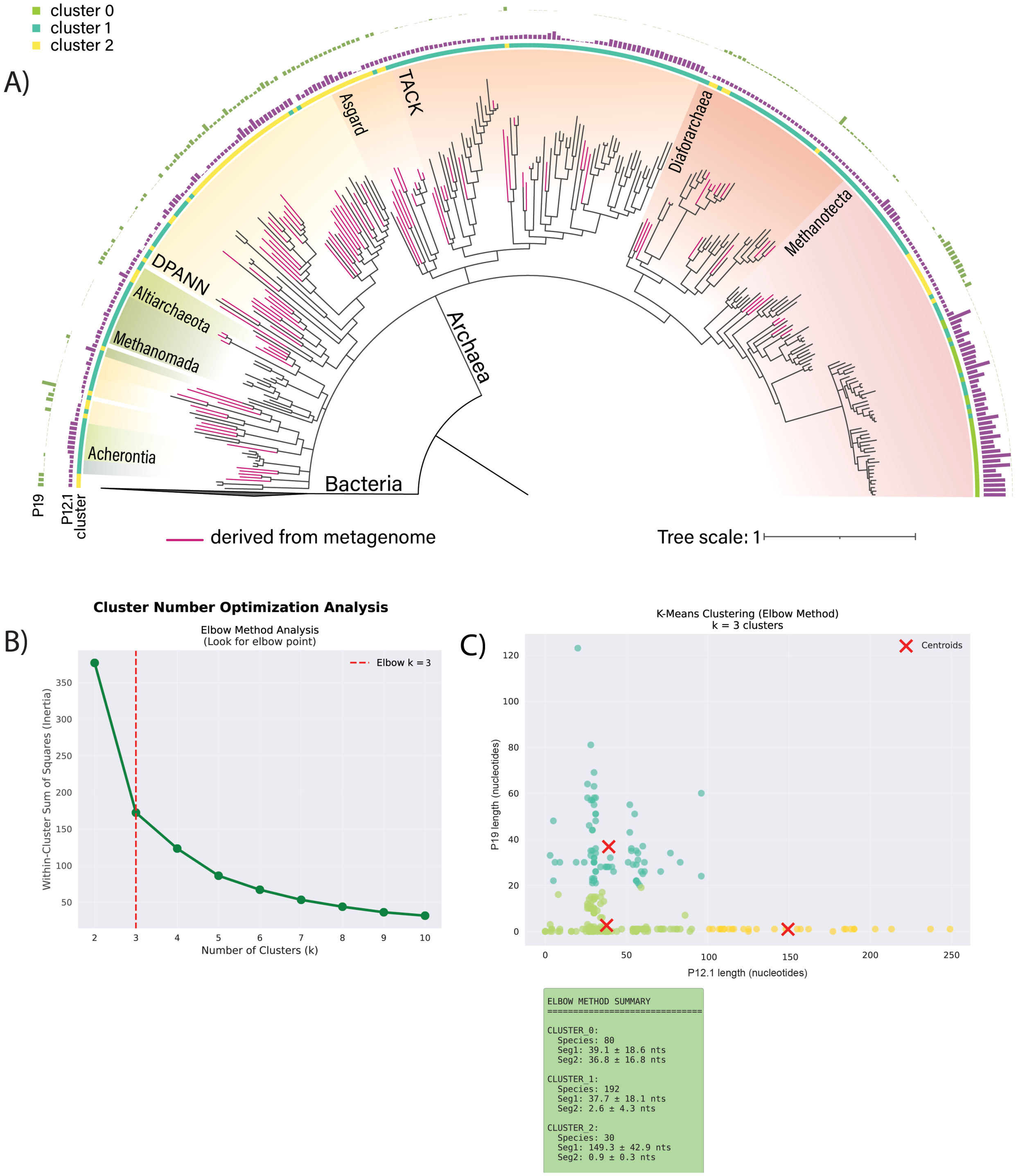

Figure S11. Size distribution of variable elements, the length of insertions within P12 and the length of P19 over archaeal phylogeny. A) P12.1 (purple bars) and P19 (light green bars) mapped onto a phylogenetic tree from Moody (1) representing the evolution of archaea. The P12.1/P19 length distribution was subjected to clustering analysis B) using the elbow method, yielding the number of clusters k=3, and C) analyzed using the K-means method.

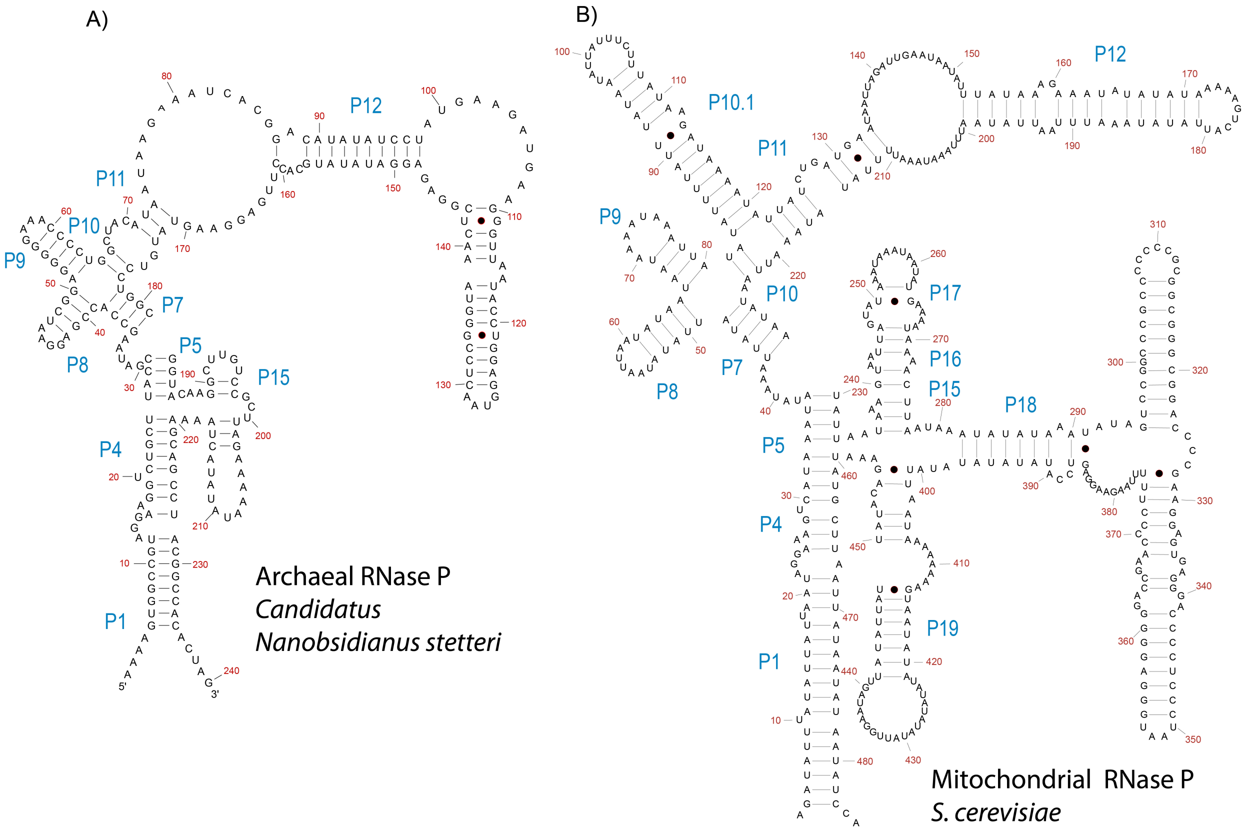

Figure S12. Revised secondary structures of reduced RPR from A) archaeal *Candidatus Nanobsidianus stetteri* (current study, Figshare File S6) and B) mitochondria of the fungus *S. cerevisiae* (13). Both RPRs reveal deletions of P3 and the 3′ portion of P2 that led to a loss of the P2 pseudoknot, an important but not essential element of RNP architecture. Helical elements are labeled in blue; nucleotides are labeled in maroon.

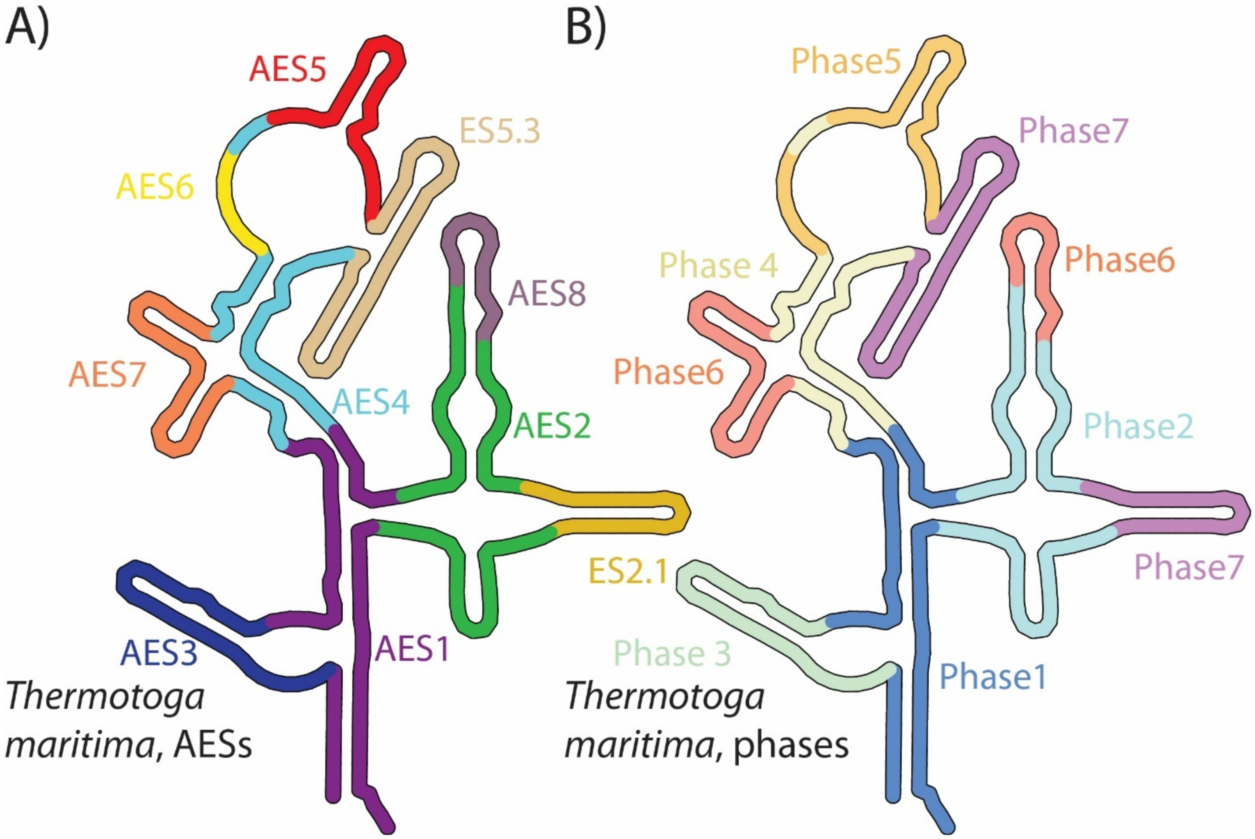

Figure S13. Schematic representation of secondary structures of RNase P RNA from bacterial *Thermotoga maritima* partitioned into A) AESs and B) evolutionary phases (33). Each AES and phase are uniquely colored such that the color boundaries represent the boundaries between the elements. The detailed relationship between AESs and phases is given in Table S3. This figure helps relate the AES representations (Figures 4 and 5) and integrated timeline (Figure 6).

Table S1. Experimental structures used to build the accretion model of RNase P evolution

Domain	*Genus species*	Components present	Res. (Å)	PDB ID	Method	Reference
Bacteria	*Thermotoga maritima*	Holoenzyme, tRNA, leader	4.2	3Q1R	X-ray	(18)
		Holoenzyme, tRNA	3.8	3Q1Q	X-ray	(18)
	Thermus thermophilus	Specificity domain	2.9	1U9S	X-ray	(19)
	Bacillus subtilis	Specificity domain	3.2	1NBS	X-ray	(20)
	Geobacillus stearothermophilus	Catalytic with partial specificity domain	3.33	2A64	X-ray	(21)
		Catalytic domain	3.6	3DHS	X-ray	(22)
Archaea	*Methanocaldococcus jannaschii*	Holoenzyme pre-tRNA	4.6	6K0B	Cryo-EM	(23)
		Holoenzyme	4.3	6K0A	Cryo-EM	(23)
Eukarya	*Homo sapiens*	Holoenzyme pre-tRNA	3.7	6AHU	Cryo-EM	(24)
	Homo sapiens	Holoenzyme	3.9	6AHR	Cryo-EM	(24)
	Saccharomyces cerevisiae	Holoenzyme pre-tRNA	3.5	6AH3	Cryo-EM	(25)
		Holoenzyme	3.5	6AGB	Cryo-EM	(25)

Table S2. Correspondence between helical elements of RPR and ESs/AESs*.

AES	Helices	Phases	Expansion Segments	Helices	Phases
AES1	P1, P4, P5, P6(5')	1	ES1.1	P5.1	7
AES2	P2(3'), P15, P16, P19	2	ES2.1	P18	7
			ES2.1.1	P18.0 (P15.1**)	7
AES3	P2(5'), P3	3	ES3.1	P3a	7
AES4	P7, P10, P11	4	ES4.1	P7.1	7
			ES4.2	P10.1	7
AES5	P12	5	ES5.1	P12a	7
			ES5.2	P12.a	7
			ES5.2.1	P12b	7
			ES5.3	P13, P14	7
AES6	None (T motif*)	5	None	NA	
AES7	P8, P9	6	ES7.1	None	7
AES8	P6(3'), P17	6	None	NA	

*Based on 3D structures.

Table S3. Stepwise accretion process modeled by ancestral expansion segments (AESs) extracted from 3D structures of a bacterial RPR of *T. maritima* (PDB id: 3Q1Q, chain A) and bacterial RPR of *G. stearothermophilus* (2A64, chain A), when explicitly mentioned. AESs are colored according to the scheme in Fig 4a, Main text. Original helix numbers and nucleotide numbers are also provided.

**AES trunk/** *AES branch*	**Helices trunk/** *Helices branch*	**Nucleotides trunk//** *Nucleotides branch*	3-Dimensional View
**Phase 1**			
**1**	**P1, P4, P5, P6(5')**	**(1-11; 46-65; 217-221; 324-347)**	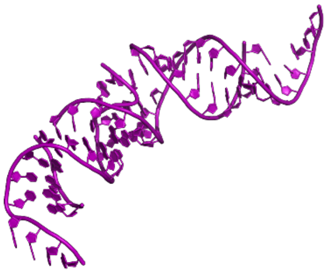
**Phase 2**			
**1**/*2*	**P1, P4, P5, P6(5')/** *P2(3'), P15, P16, P19*	**(1-11; 46-65; 217-221; 324-347)**//*(222-240; 259-273) & 2A64: (330-390))*	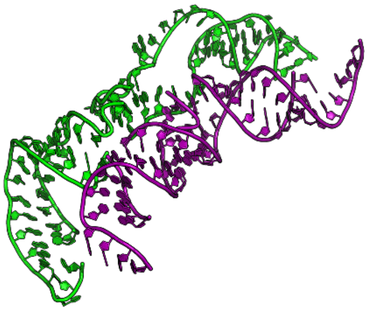
Phase 3			
**1&2**/*3*	**P1, P4, P5, P6(5'), P2(3'), P15, P16, P19/** *P2(5'), P3*	**(1-11; 46-65; 217-221; 324-347) & (222-240; 259-273) & 2A64: (330-390)**//*(12-45)*	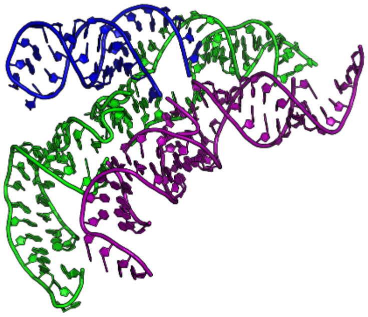
Phase 4			

**1**/*4*	**P1, P4, P5, P6(5')**/ *P7, P10, P11*	**(1-11; 46-65; 217-221; 324-347)**// *(66-72; 102-111; 119-121; 202-216)*	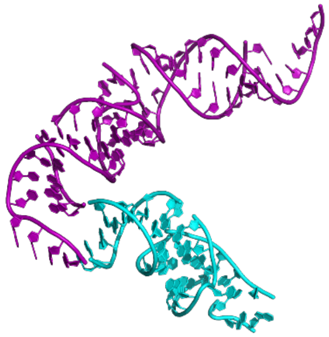
Phase 5			
**4**/*5*	**P7, P10, P11**/*P12*	**(66-72; 102-111; 119-121; 202-216)**// *(122-133; 143-159)*	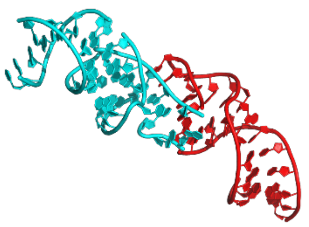
**4&5**/*6*	**P7, P10, P11, *P12*/** *T-motif*	**(66-72; 102-111; 119-121; 202-216) & (122-133; 143-159)**/// *(112-118)*	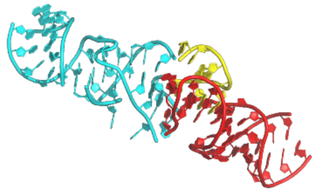
Phase 6.			
**4**/*7*	**P7, P10, P11**/*P8, P9*	**(66-72; 102-111; 119-121; 202-216)**//*(73-101)*	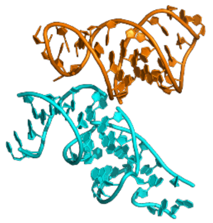
**1&2**/*8*	**P1, P4, P5, P6(5'), P2(3'), P15, P16, P19//** *P6(3'), P17*	**(1-11; 46-65; 217-221; 324-347) & (222-240; 259-273) & 2A64: (330-390)**//*(241-258)*	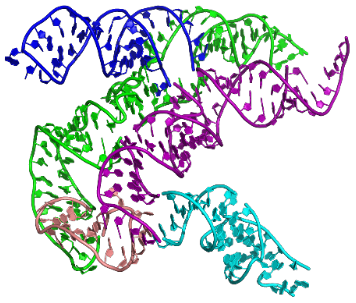

Table S4. Definitions of post LUCA linage specific expansion segments of bacterial, archaeal, and eukaryotic RPRs used in the current study. ES are colored according to the scheme in Fig 4a, Mian Text. Original helix numbers and nucleotide numbers are also provided.

**AES trunk/** *ES branch*	**Helices trunk/** *Helices branch*	**Nucleotides trunk/** *Nucleotides branch (type)*	3-Dimensional View
Geobacillus stearothermophilus, 2A64, Chain A			
**1/***1.1*	**P1, P4, P5/***P5a*	**(1-14; 46-62; 81-83;250-255; 391-414)//***(63-80)*	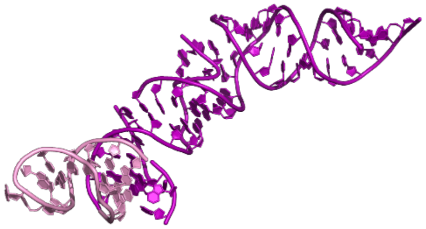
Geobacillus stearothermophilus, 2A64, Chain A			
**2** /*2.1 2.1.1*	**P2(3'), P15,** /*P18.0, P18*	**(256-277; 330-390)//***(278-329)*	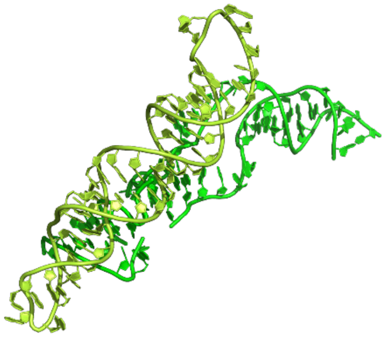
Thermotoga maritima, 3Q1Q, Chain A			
**2/***2.2*	**P2(3'), P15, P16, P17/**P18	**(222-240; 259-273; 298-323)**//*(274-297)*	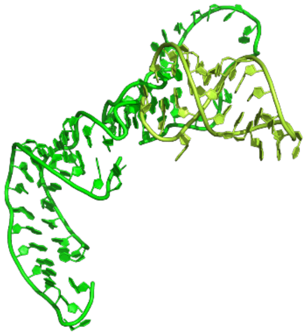
Homo sapiens, 6AHU, chain A			
**3/***3.1*	**P2(5'), P3/P3a**	**(17-34; 59-73)**//*(35-58)*	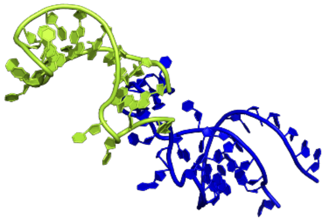
Geobacillus stearothermophilus, 2A64, chain A			

**4/***4.2*	**P7, P10, P11/P10a**	**(84-91; 139-141; 190-194; 236-249)***//(142-189)*	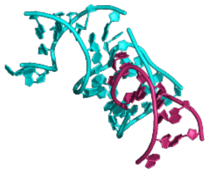
Methanocaldococcus jannaschii, 6K0B, chain X			
**5/***5.1*	**P12/***P12a*	***(104-123; 147-166)//****(124-146)*	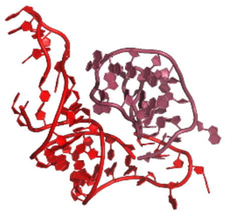
Homo sapiens, 6AHU, chain A			
**5/***5.2+ 5.2.1*	**P12/***P12a+ 12b*	***(152-170; 228-247)//****(171-227)*	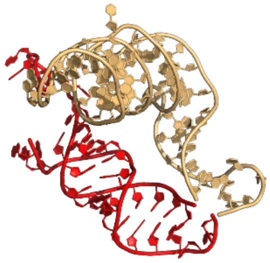
Thermotoga maritima, 3Q1Q, chain A			
**4+5/ 5.3**	**P7, P10, P11, P12/** *P13, P14*	**(66-72;102-111; 119-121; 122-133; 143-159;202-216)***//* *(160-201)*	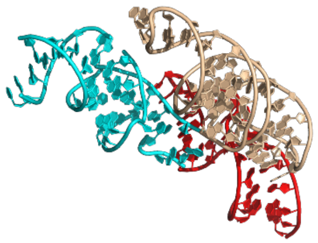

Table S5. List of the secondary structures remodeled from study by Brown (55), depicting P4 as an element of the secondary structure and P2 as a pseudoknot. The secondary structures are given in Figshare File S6.

Bacterial Species	Type	Archaeal and Eukaryotic Species	Type
Ralstonia eutropha	A2	*Methanothermobacter thermautotrophicus*	A
Chlamydia trachomatis	A3	*Pyrobaculum aerophilum*	T
Synechococcus elongatus	A4	*Thermoplasmata archaeon*	
Chlorobium limicola	A5	*Methanocaldococcus jannaschii*	M
Thermomicrobium roseum	C	*Thermoplasma volcanium GSS1*	N/A
Mycoplasma hyopneumoniae	B2	*Candidatus Nanobsidianus stetteri*	N/A
Mycoplasma ferrmentans	B3	*Homo sapiens*	
Geobacillus stearothermophilus	B1	Mitochondrial Pichia canadensis	
Thermotoga maritima	A1		
Escherichia coli	A		

Table S6. Summary of A-minor interactions among AESs and between AESs and ESs for archaeal and bacterial species.

**A-minor acceptor helix (AES)**	**A-minor donor nucleotide (AES/ES)**		
	T. maritima (*3Q1Q, chain A*)	*G. stearothermophilus* (*2A64, chain A*)	*M. jannaschii* (*6K0B, chain X*)
P1 (AES 1)	G95 (AES 7)		A75(AES5)
P1 (AES 1)	A96 (AES 7)		A77(AES5)
P4 (AES 1)	A79 (AES 7)	A99(AES5)	
P4 (AES 1)	A80 (AES 7)	A100(AES5)	
P5(AES1)	A223 (A214*) (AES 2)	A257(AES2)	A192(AES2)
TΨC stem (tRNA)	A207 (A198*) (AES 4)	A137(AES7)** A240(AES4)**	
P12 (AES 5)	A168 (A159*) (ES 5.3)		
P12 (AES 5)	A169 (A160*) (ES 5.3)		
P8 (AES 7)	A190 (A181*) (ES 5.3)		
P8 (AES 7)	A191 (A182*) (ES 5.3)		
P8 (AES 7)	A287 (A278*) (ES 2.1)		
P8 (AES 7)	A288 (A279*) (ES 2.1)		
P7 (AES 4)		A187 (ES 4.2)	
P10 (AES 4)		A188 (ES 4.2)	

*Numbering from the wild-type RNase P of *T. maritima*

**Inferred from the superimposition with tRNA from *T. maritima* (3Q1Q, chain C)

References

1. Moody ERR, Mahendrarajah TA, Dombrowski N, Clark JW, Petitjean C, Offre P, Szöllősi GJ, Spang A, & Williams TA (2022) An estimate of the deepest branches of the tree of life from ancient vertically evolving genes. *eLife* **11**: e66695.
2. Nawrocki EP & Eddy SR (2013) Infernal 1.1: 100-fold faster RNA homology searches. *Bioinformatics* **29**: 2933-2935.
3. Eddy SR & Durbin R (1994) RNA sequence analysis using covariance models. *Nucleic Acids Res* **22**: 2079-2088.
4. Burge SW, Daub J, Eberhardt R, Tate J, Barquist L, Nawrocki EP, Eddy SR, Gardner PP, & Bateman A (2012) Rfam 11.0: 10 years of RNA families. *Nucleic Acids Res* **41**: D226-D232.
5. Griffiths-Jones S, Bateman A, Marshall M, Khanna A, & Eddy SR (2003) Rfam: An RNA family database. *Nucleic Acids Res* **31**: 439-441.
6. Garcia-Martin JA, Dotu I, & Clote P (2015) Rnaifold 2.0: A web server and software to design custom and rfam-based RNA molecules. *Nucleic Acids Res* **43**: W513-W521.
7. Letunic I & Bork P (2024) Interactive tree of life (itol) v6: Recent updates to the phylogenetic tree display and annotation tool. *Nucleic Acids Res* **52**: W78-W82.
8. Meade C, Banerjee B, Yang Y, Suri A, Hoksza D, Williams LD, & Petrov AS (2026) Exornata: A web-based editor for the visualization and editing of RNA secondary structures. *J Mol Biol* 169632.
9. Sweeney BA, Hoksza D, Nawrocki EP, Ribas CE, Madeira F, Cannone JJ, Gutell R, Maddala A, Meade CD, Williams LD, Petrov AS, Chan PP, Lowe TM, Finn RD, & Petrov AI (2021) R2DT is a framework for predicting and visualising RNA secondary structure using templates. *Nat Commun* **12**: 3494.
10. Mccann H, Meade CD, Williams LD, Petrov AS, Johnson PZ, Simon AE, Hoksza D, Nawrocki EP, Chan PP, Lowe TM, Ribas CE, Sweeney BA, Madeira F, Anyango S, Appasamy SD, Deshpande M, Varadi M, Velankar S, Zirbel CL, Naiden A, Jossinet F, & Petrov AI (2025) R2DT: A comprehensive platform for visualizing RNA secondary structure. *Nucleic Acids Res* **53**.
11. Ellis JC & Brown JW (2009) The RNase P family. *RNA Biol* **6**: 362-369.
12. Lu X-J, Bussemaker HJ, & Olson WK (2015) DSSR: An integrated software tool for dissecting the spatial structure of RNA. *Nucleic Acids Res* **43**: e142-e142.
13. Seif ER, Forget L, Martin NC, & Lang BF (2003) Mitochondrial RNase P RNAs in ascomycete fungi: Lineage-specific variations in RNA secondary structure. *RNA* **9**: 1073-1083.
14. Hollingsworth MJ & Martin NC (1986) RNase P activity in the mitochondria of Saccharomyces cerevisiae depends on both mitochondrion and nucleus-encoded components. *Mol Cell Biol* **6**: 1058-1064.
15. Podar M, Makarova KS, Graham DE, Wolf YI, Koonin EV, & Reysenbach AL (2013) Insights into archaeal evolution and symbiosis from the genomes of a nanoarchaeon and its inferred crenarchaeal host from obsidian pool, yellowstone national park. *Biol Direct* **8**: 9.

16. Munson-McGee JH, Field EK, Bateson M, Rooney C, Stepanauskas R, & Young MJ (2015) Nanoarchaeota, their sulfolobales host, and nanoarchaeota virus distribution across yellowstone national park hot springs. *Appl Environ Microbiol* **81**: 7860-7868.
17. Berman HM, Westbrook J, Feng Z, Gilliland G, Bhat TN, Weissig H, Shindyalov IN, & Bourne PE (2000) The protein data bank. *Nucleic Acids Res* **28**: 235-242.
18. Reiter NJ, Osterman A, Torres-Larios A, Swinger KK, Pan T, & Mondragón A (2010) Structure of a bacterial ribonuclease P holoenzyme in complex with tRNA. *Nature* **468**: 784-789.
19. Krasilnikov AS, Xiao Y, Pan T, & Mondragón A (2004) Basis for structural diversity in homologous RNAs. *Science* **306**: 104-107.
20. Krasilnikov AS, Yang X, Pan T, & Mondragón A (2003) Crystal structure of the specificity domain of ribonuclease P. *Nature* **421**: 760-764.
21. Kazantsev AV, Krivenko AA, Harrington DJ, Holbrook SR, Adams PD, & Pace NR (2005) Crystal structure of a bacterial ribonuclease P RNA. *Proc Natl Acad Sci USA* **102**: 13392-13397.
22. Kazantsev AV, Krivenko AA, & Pace NR (2009) Mapping metal-binding sites in the catalytic domain of bacterial RNase P RNA. *RNA* **15**: 266-276.
23. Wan F, Wang Q, Tan J, Tan M, Chen J, Shi S, Lan P, Wu J, & Lei M (2019) Cryo-electron microscopy structure of an archaeal ribonuclease P holoenzyme. *Nat Commun* **10**: 1-13.
24. Wu J, Niu S, Tan M, Huang C, Li M, Song Y, Wang Q, Chen J, Shi S, & Lan P (2018) Cryo-EM structure of the human ribonuclease P holoenzyme. *Cell* **175**: 1393-1404. e1311.
25. Lan P, Tan M, Zhang Y, Niu S, Chen J, Shi S, Qiu S, Wang X, Peng X, & Cai G (2018) Structural insight into precursor tRNA processing by yeast ribonuclease P. *Science* **362**.
26. Delano WL (2002) Pymol: An open-source molecular graphics tool. *CCP4 Newsletter on Protein Crystallogr* **40**: 82-92.
27. Shindyalov IN & Bourne PE (1998) Protein structure alignment by incremental combinatorial extension (CE) of the optimal path. *Protein Eng* **11**: 739-747.
28. Finn RD, Clements J, & Eddy SR (2011) Hmmer web server: Interactive sequence similarity searching. *Nucleic Acids Res* **39**: W29-37.
29. Katoh K & Standley DM (2013) MAFFT multiple sequence alignment software version 7: Improvements in performance and usability. *Mol Biol Evol* **30**: 772-780.
30. Frickey T & Lupas A (2004) Clans: A java application for visualizing protein families based on pairwise similarity. *Bioinformatics* **20**: 3702-3704.
31. Steinegger M, Meier M, Mirdita M, Vöhringer H, Haunsberger SJ, & Söding J (2019) HH-suite3 for fast remote homology detection and deep protein annotation. *BMC Bioinformatics* **20**: 473.
32. Petrov AS, Bernier CR, Hsiao C, Norris AM, Kovacs NA, Waterbury CC, Stepanov VG, Harvey SC, Fox GE, Wartell RM, Hud NV, & Williams LD (2014) Evolution of the ribosome at atomic resolution. *Proc Natl Acad Sci USA* **111**: 10251-10256.

33. Petrov AS, Gulen B, Norris AM, Kovacs NA, Bernier CR, Lanier KA, Fox GE, Harvey SC, Wartell RM, Hud NV, & Williams LD (2015) History of the ribosome and the origin of translation. *Proc Natl Acad Sci USA* **112**: 15396–15401.
34. Guerrier-Takada C, Gardiner K, Marsh T, Pace N, & Altman S (1983) The RNA moiety of ribonuclease P is the catalytic subunit of the enzyme. *Cell* **35**: 849-857.
35. Robertson HD, Altman S, & Smith JD (1972) Purification and properties of a specific Escherichia coli ribonuclease which cleaves a tyrosine transfer ribonucleic acid precursor. *J Biol Chem* **247**: 5243-5251.
36. James BD, Olsen GJ, Liu J, & Pace NR (1988) The secondary structure of ribonuclease P RNA, the catalytic element of a ribonucleoprotein enzyme. *Cell* **52**: 19-26.
37. Fox GE & Woese CR (1975) 5S RNA secondary structure. *Nature* **256**: 505-507.
38. Pace NR, Smith DK, Olsen GJ, & James BD (1989) Phylogenetic comparative analysis and the secondary structure of ribonuclease P RNA — a review. *Gene* **82**: 65-75.
39. Haas ES, Morse DP, Brown JW, Schmidt FJ, & Pace NR (1991) Long-range structure in ribonuclease P RNA. *Science* **254**: 853-856.
40. Zarrinkar PP, Wang J, & Williamson JR (1996) Slow folding kinetics of RNase P RNA. *RNA* **2**: 564-573.
41. Loria A & Pan T (1996) Domain structure of the ribozyme from eubacterial ribonuclease P. *RNA* **2**: 551-563.
42. Massire C, Jaeger L, & Westhof E (1998) Derivation of the three-dimensional architecture of bacterial ribonuclease P RNAs from comparative sequence analysis. *J Mol Biol* **279**: 773-793.
43. Rivas E (2020) RNA structure prediction using positive and negative evolutionary information. *PLoS Comp Biol* **16**: e1008387.
44. Smit S, Rother K, Heringa J, & Knight R (2008) From knotted to nested RNA structures: A variety of computational methods for pseudoknot removal. *RNA* **14**: 410-416.
45. Torres-Larios A, Swinger KK, Krasilnikov AS, Pan T, & Mondragón A (2005) Crystal structure of the RNA component of bacterial ribonuclease P. *Nature* **437**: 584-587.
46. Kalvari I, Nawrocki EP, Ontiveros-Palacios N, Argasinska J, Lamkiewicz K, Marz M, Griffiths-Jones S, Toffano-Nioche C, Gautheret D, & Weinberg Z (2021) Rfam 14: Expanded coverage of metagenomic, viral and microRNA families. *Nucleic Acids Res* **49**: D192-D200.
47. Masquida B & Westhof E (2011) RNase P: At last, the key finds its lock. *RNA* **17**: 1615-1618.
48. Phan HD, Lai LB, Zahurancik WJ, & Gopalan V (2021) The many faces of RNA-based RNase P, an RNA-world relic. *Trends Biochem Sci* **46**: 976-991.
49. Kawano S, Nakashima T, Kakuta Y, Tanaka I, & Kimura M (2006) Crystal structure of protein Ph1481p in complex with protein Ph1877p of archaeal RNase P from pyrococcus horikoshii OT3: Implication of dimer formation of the holoenzyme. *J Mol Biol* **357**: 583-591.
50. Wilson RC, Bohlen CJ, Foster MP, & Bell CE (2006) Structure of Pfu Pop5, an archaeal RNase P protein. *Proc Natl Acad Sci USA* **103**: 873-878.

51. Petrov AS, Bernier CR, Gulen B, Waterbury CC, Hershkovits E, Hsiao C, Harvey SC, Hud NV, Fox GE, Wartell RM, & Williams LD (2014) Secondary structures of rRNAs from all three domains of life. *PLoS One* **9**: e88222.
52. Hug LA, Baker BJ, Anantharaman K, Brown CT, Probst AJ, Castelle CJ, Butterfield CN, Hernsdorf AW, Amano Y, Ise K, Suzuki Y, Dudek N, Relman DA, Finstad KM, Amundson R, Thomas BC, & Banfield JF (2016) A new view of the tree of life. *Nat Microbiol* **1**: 16048.
53. Varadi M, Bertoni D, Magana P, Paramval U, Pidruchna I, Radhakrishnan M, Tsenkov M, Nair S, Mirdita M, Yeo J, Kovalevskiy O, Tunyasuvunakool K, Laydon A, Žídek A, Tomlinson H, Hariharan D, Abrahamson J, Green T, Jumper J, Birney E, Steinegger M, Hassabis D, & Velankar S (2023) Alphafold protein structure database in 2024: Providing structure coverage for over 214 million protein sequences. *Nucleic Acids Res* **52**: D368-D375.
54. Ahmad S, Jose da costa gonzales L, Bowler-Barnett Emily h, Rice Daniel l, Kim M, Wijerathne S, Luciani A, Kandasaamy S, Luo J, Watkins X, Turner E, Martin Maria j, & Consortium TU (2025) The UniProt website api: Facilitating programmatic access to protein knowledge. *Nucleic Acids Res* **53**: W547-W553.
55. Brown JW (1998) The ribonuclease P database. *Nucleic Acids Res* **26**: 351-352.
